# Copper(ii) complex-decorated ZrFe_2_O_4_ nanoparticles as a recyclable magnetic nanocatalyst for synthesis of N-containing heterocycles†

**DOI:** 10.1039/d4na01058b

**Published:** 2025-05-12

**Authors:** Tara Miladi, Masoomeh Norouzi

**Affiliations:** a Department of Chemistry, Faculty of Science, Ilam University P.O. Box 69315516 Ilam Iran m.norozi@ilam.ac.ir norouzi_88organic@yahoo.com

## Abstract

In this study, a novel and magnetic nanocatalyst [ZrFe_2_O_4_@SiO_2_@GLYMO-oPD-Cu(ii)] was developed by immobilization of an *o*-phenylenediamine–copper complex on ZrFe_2_O_4_@SiO_2_ nanoparticles. The as-prepared nanocatalyst was identified by physicochemical techniques such as FT-IR, BET, SEM, VSM, XRD, TEM, TGA, DSC, ICP EDX, and EDX elemental mapping. This nanocomposite exhibited remarkable efficiency in promoting the green synthesis of nitrogen-rich heterocycles, including 5-substituted 1*H*-tetrazoles and 2,3-dihydroquinazolin-4(1*H*)-one derivatives, through click and cyclization reactions, respectively. The reactions were conducted under mild conditions, affording high yields in short reaction times. Furthermore, the catalyst demonstrated excellent stability and reusability, with minimal copper leaching observed across multiple catalytic cycles.

## Abstract 2

A novel, magnetically separable copper(ii) complex was synthesized and effectively catalyzed the green synthesis of nitrogen-rich heterocycles under mild conditions with high yields, excellent stability, and recyclability.

## Graphical abstract

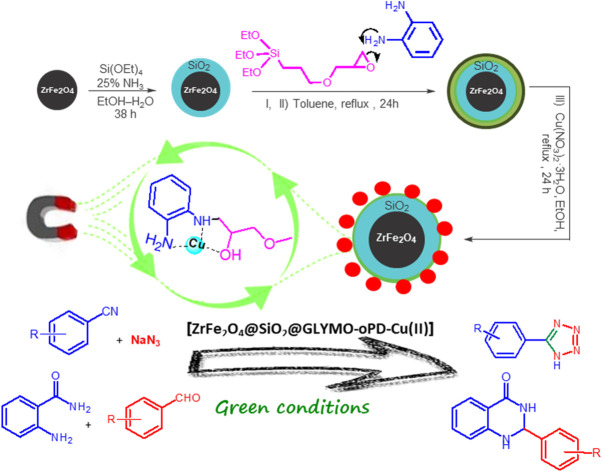

## Introduction

With the advent of green chemistry, clean and efficient chemical reactions have become the inevitable development trend of the chemical industry. At present, one of the strategies to achieve this goal is to perform chemical reactions in the presence of a catalyst.^1,2^ In the field of catalyst knowledge, achieving a high level of activity and selectivity to carry out a catalytic reaction is a challenging issue.^3–5^ Heterogeneous catalysts play a significant role in promoting environmentally friendly and low-cost protocols in the synthesis of organic compounds.^6,7^

The special advantage of such catalysts is the ability to reuse and recycle their materials compared to other homogeneous organic and inorganic catalysts.^8,9^ Nanocatalysts act as an efficient and suitable linker between heterogeneous and homogeneous catalysts.^8,10^

Among different nanomaterials, magnetite nanoparticles (MNPs) have received increasing attention in chemistry and other fields due to their numerous and unique features, including low preparation cost, large-scale production capability, simple synthesis method, and mechanical and thermal stability.^11–13^ Also, their most important property, namely paramagnetism, enables their easy and efficient separation in the separation of heterogeneous catalysts, and they can be easily separated from the reaction mixture using an external magnet.^11,14^

In this context, the development of new and reliable heterogeneous catalytic supports is a widely researched topic in the fields of industrial and catalysis science.^15,16^ ZrFe_2_O_4_ MNPs, also known as zirconium ferrite, is a promising material with potential for this purpose. These MNPs possess several advantageous properties, including low cost, availability, thermal stability, good binding to organic molecules, high concentration of surface hydroxyl groups, small size, a high specific surface area, thermal stability, optical properties, low density, and electrical properties. As such, ZrFe_2_O_4_ MNPs are the ideal candidate for use as a novel catalytic support in the immobilization of homogeneous catalysts.^17–23^

Over the past decade, the synthesis of nitrogen-rich heterocyclic compounds has garnered immense interest due to their sustainable nature.^24–26^ Tetrazoles are a class of doubly unsaturated aromatic heterocycles with four nitrogen atoms, possessing unique properties including high nitrogen content, a large dipole moment, high energy content, and strong hydrogen bonding capabilities.^27,28^ These properties, combined with their excellent chemical stability, make them valuable bioisosteres for *cis*-amide bonds, leading to diverse applications in pharmaceuticals and materials science.^8,26^ Due to their synthetic nature, laboratories around the world have initiated efforts to synthesize tetrazoles to further explore their chemistry and potential functions.^8,29–35^

2,3-Dihydroquinazolin-4(1*H*)-ones are a type of N-heterocyclic compound that have attracted significant attention from researchers due to their wide-ranging biological activities.^36–38^ These compounds, characterized by their unique chemical structure, have shown promise in various therapeutic areas, including cancer treatment, inflammation reduction, and neurological disorders.^36,37,39^ The incorporation of the quinazolinone core, a privileged structure known for its ability to bind to multiple receptors, enhances the potential of these compounds to interact with biological targets effectively.^40–42^ Consequently, the synthesis of quinazolinone-based heterocycles has become a focal point in medicinal chemistry, with numerous synthetic strategies developed to access these valuable molecules.^42–47^

Recognizing the environmental impact of chemical synthesis, in this work we introduce a novel, eco-friendly, and recyclable heterogeneous catalyst, [ZrFe_2_O_4_@SiO_2_@GLYMO-oPD-Cu(ii)], for the synthesis of 5-substituted 1*H*-tetrazoles and 2,3-dihydroquinazolin-4(1*H*)-ones. This catalyst aligns with green chemistry principles by minimizing waste generation, reducing energy consumption, and promoting the use of benign reagents. Its reusability further enhances its sustainability profile, making it a promising candidate for industrial applications.

## Experimental

### Materials and methods

The chemicals used in this study were sourced from Merck and Aldrich and utilized without purification. FTIR analysis (VRTEX 70 model BRUKER FT-IR, 400–4000 cm^−1^) identified functional group compositions. XRD patterns were obtained from 2*θ* = 10–80° (Philips X'Pert Pro, 1.5405 Å) to determine nanoparticle crystallography. Thermogravimetric analysis was conducted on a thermal analyzer NETZSCH STA 449 F3 Jupiter from 25 °C to 800 °C at a heating rate of 10 °C min^−1^, in an air atmosphere. The surface morphology of the catalyst was surveyed using an FESEM-TESCAN MIRA3. Additionally, an SEM-integrated EDAX and EDS-mapping system was employed to assess the elemental composition of the supported catalyst. Also, the magnetic properties of the nanocatalyst were measured with the help of a vibrating sample magnetometer (VSM device (LBKFB) from Magnetic Kavir Kashan). TEM was performed using a Zeiss microscope at a voltage of 100 kV. The BET analysis was carried out using a BELSORP MINI II instrument.

### Typical procedure for synthesis of [ZrFe_2_O_4_@SiO_2_@GLYMO-oPD-Cu(ii)] MNPs

Initially, a mixture of 3-glycidoxypropyltriethoxysilane (2.78 g, 10 mmol) and *o*-phenylenediamine (1.08 g, 10 mmol) was added to a round-bottom flask containing 25 mL of dry toluene. The mixture was refluxed for 24 hours. Subsequently, the reaction mixture was added to a well-dispersed suspension of ZrFe_2_O_4_@SiO_2_ MNPs (2 g) in 25 mL of toluene, and the reflux was continued for an additional 24 hours. The synthesized ZrFe_2_O_4_@SiO_2_@GLYMO-oPD nanoparticles were magnetically filtered and washed thoroughly with ethanol to remove unreacted substrates, and dried in an oven at 80 °C. Finally, 1 g of ZrFe_2_O_4_@SiO_2_@GLYMO-oPD nanoparticles was dispersed in 50 mL of ethanol by sonication for 30 minutes. Then, 2.5 mmol of Cu(NO_3_)_2_·3H_2_O was added to the reaction mixture and stirred at 80 °C for 24 hours. The final [ZrFe_2_O_4_@SiO_2_@GLYMO-oPD-Cu(ii)] complex was magnetically separated, washed thoroughly with water and ethanol to remove unreacted copper, and dried in an oven at 80 °C for 4 h.

### General procedure for synthesis of 5-substituted 1*H*-tetrazoles using the [ZrFe_2_O_4_@SiO_2_@GLYMO-oPD-Cu(ii)] complex

A 25 mL round-bottom flask was charged with a mixture of ZrFe_2_O_4_@SiO_2_@GLYMO-oPD-Cu(ii) (20 mg), benzonitrile (1 mmol), sodium azide (NaN_3_, 1.2 mmol) and 2 mL of PEG-400 as the solvent. The reaction mixture was stirred in a pre-heated sand bath at 120 °C. After complete consumption of the starting material (checked by TLC), the mixture was diluted with 15 mL of ethyl acetate and water, and the catalyst was magnetically separated. The mixture was then transferred to a separatory funnel and acidified with 10 mL of 4 N hydrochloric acid (HCl) solution. The organic phase was dried over sodium sulfate, the solvent was evaporated, and further purification was achieved using preparative silica gel TLC plates.

### General procedure for synthesis of 2,3-dihydroquinazolin-4(1*H*)-ones using the [ZrFe_2_O_4_@SiO_2_@GLYMO-oPD-Cu(ii)] complex

A 25 mL round-bottom flask was charged with a mixture of ZrFe_2_O_4_@SiO_2_@GLYMO-oPD-Cu(ii) (35 mg), 2-aminobenzamide (1 mmol), aldehydes (1 mmol) and 3 mL of ethanol as the solvent. The reaction mixture was stirred under reflux conditions to ensure that the reaction was completed (monitored by TLC). Then the catalyst was magnetically separated from the reaction mixture. The mixture was diluted with 15 mL of hot ethanol, and the catalyst was magnetically separated. The crude solid product was then purified by recrystallization in ethanol.

### Spectral data

5-Phenyltetrazole (Table 2, entry 1): ^1^H NMR (250 MHz, DMSO-d_6_) *δ*: 16.83 (b, 1H, N–H), 8.03–8.02 (d, *J* = 8 Hz, 2H, CHAr), 7.60 (t, *J* = 7.9 Hz, 3H, CHAr).

5-*p*-Tolyl-1H-tetrazole (Table 2, entry 2): ^1^H NMR (250 MHz, DMSO-d_6_) *δ*: 16.74 (s, 1H, NH), 7.93–7.89 (d, *J* = 8 Hz, 2H, CHAr), 7.41–7.38 (d, *J* = 8 Hz, 2H, CHAr), 2.33 (s, 3H, CH_3_).

5-(2-Fluoro-phenyl)-1*H*-tetrazole (Table 2, entry 5): ^1^H NMR (250 MHz, DMSO-d_6_) *δ*: 17.07 (b, 1H, N–H), 8.34 (s, 1H, CHAr), 7.97–7.93 (d, *J* = 8 Hz, 1H, CHAr), 7.87–7.81 (m, 2H, CHAr)

5-(4-Chloro-phenyl)-1*H*-tetrazole (Table 2, entry 7): ^1^H NMR (250 MHz, DMSO-d_6_) *δ*: 1693 (s, 1H, NH), 8.05–8.02 (d, *J* = 8 Hz, 2H, CHAr), 7.69–7.66 (d, *J* = 8 Hz, 2H, CHAr).

5-(4-Bromo-phenyl)-1*H*-tetrazole (Table 2, entry 8): ^1^H NMR (250 MHz, DMSO-d_6_) *δ*: 16.87 (b, 1H, N–H), 7.97–7.94 (d, *J* = 8 Hz, 2H, CHAr), 7.83–7.80 (d, *J* = 8 Hz, 2H, CHAr).

5-(3-Trifluoromethyl-phenyl)-1*H*-tetrazole (Table 2, entry 10): ^1^H NMR (250 MHz, DMSO-d_6_) *δ*: 8.07 (s, 2H, CHAr), 7.95–7.88 (t, *J* = 7.9 Hz, 1H, CHAr), 7.80–7.73 (t, *J* = 7.9 Hz, 1H, CHAr)

2-(1*H*-Tetrazol-5-yl)-benzonitrile (Table 2, entry 11): ^1^H NMR (250 MHz, DMSO-d_6_) *δ*: 16.93 (b, 1H, N–H), 7.80–7.75 (s, 2H, CHAr), 7.94–7.88 (d, *J* = 8 Hz, 1H, CHAr), 7.79–7.76 (m, 1H, CHAr).

2-(4-Bromo-phenyl)-2,3-dihydro-1*H*-quinazolin-4-one (Table 4, entry 2): ^1^H NMR (250 MHz, DMSO-d_6_) *δ*: 8.31 (s, 1H, NH), 7.60–7.56 (d, *J* = 7.6 Hz, 3H, CHAr), 7.43–7.40 (d, *J* = 7.4 Hz, 2H, CHAr), 7.26–712 (s, *J* = 7 Hz, 1H, CHAr), 7.12 (s, 1H, NH), 6.70–6.66 (m, 2H, CHAr), 5.73 (s, 1H, CH) ppm.

2-Phenyl-2,3-dihydro-1*H*-quinazolin-4-one (Table 4, entry 7): ^1^H NMR (250 MHz, DMSO-d_6_) *δ*: 8.26 (s, 1H, NH), 7.60–7.57 (d, *J* = 7.6 Hz, 1H, CHAr), 7.46 (s, 2H, CHAr), 7.37–7.34 (m, 3H, CHAr), 7.25–7.19 (t, *J* = 7.22 Hz, 1H, CHAr), 7.09 (s, 1H, NH), 6.74–6.62 (m, *J* = 6.68 Hz, 2H, CHAr), 5.73 (s, 1H, CH).

2-(4-Chloro-phenyl)-2,3-dihydro-1*H*-quinazolin-4-one (Table 4, entry 10): ^1^H NMR (250 MHz, DMSO-d_6_) *δ*: 8.31 (s, 1H), 7.60–7.57 (d, *J* = 7.1 Hz, 1H, CHAr), 7.50–7.41 (m, 4H, CHAr), 7.26–7.19 (t, 1H, CHAr), 7.12 (s, 1H, NH), 6.73–6.66 (t, *J* = 7.0, 2H, CHAr), 5.74 (s, 1H, CH) ppm.

## Results and discussion

**Scheme 1:**
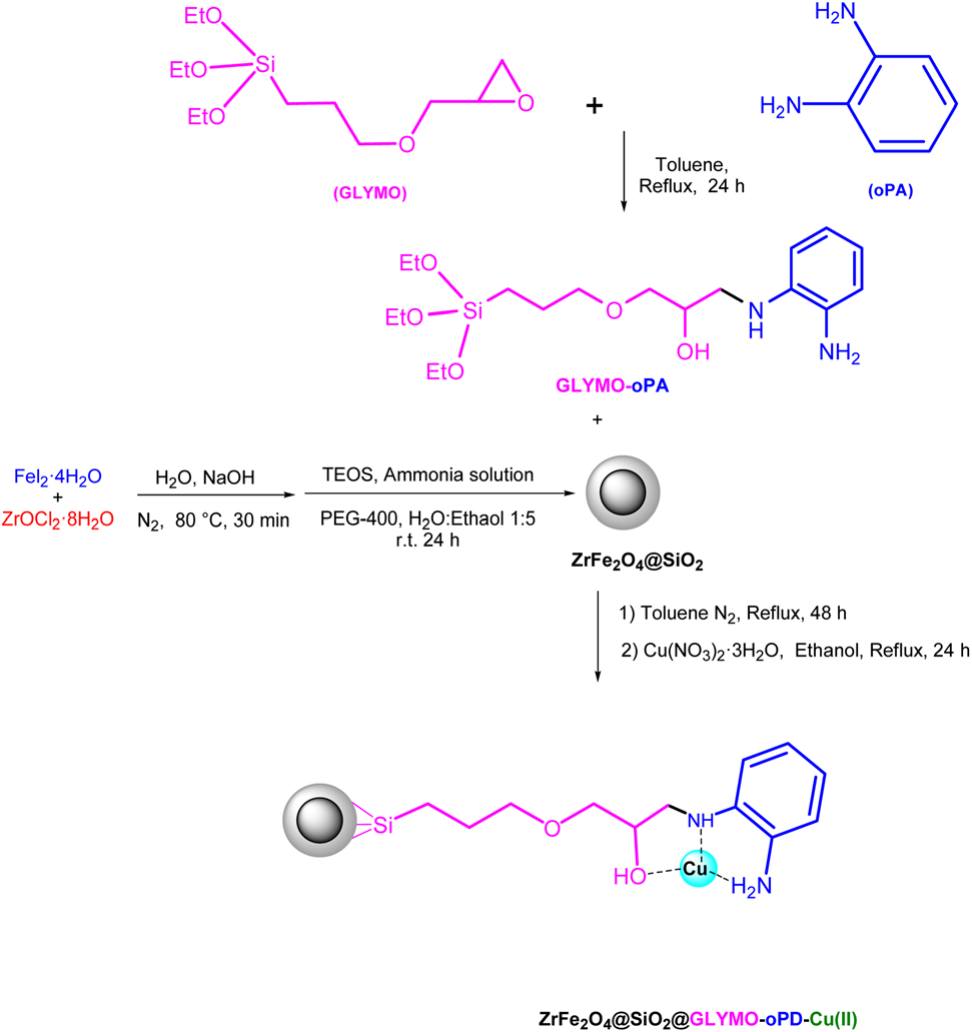
Stepwise synthesis of the [ZrFe_2_O_4_@SiO_2_@GLYMO-oPD-Cu(ii)] complex.

The core–shell structure [ZrFe_2_O_4_@SiO_2_@GLYMO-oPD-Cu(ii)] complex was successfully synthesized through a facile multi-step process. Initially, ZrFe_2_O_4_ MNPs were prepared by a precipitation reaction. Subsequently, the ZrFe_2_O_4_ MNPs were coated with an amorphous silica layer to enhance their stability and provide additional functionalization sites. Finally, a copper complex of GLYMO-oPD was immobilized onto the silica surface to obtain the desired catalyst (Scheme 1). The synthesized catalyst was characterized using various techniques, including Fourier Transform Infrared Spectroscopy (FT-IR), XRD, Thermogravimetric analysis (TGA), differential scanning calorimetry (DSC), transmission electron microscopy (TEM), energy-dispersive X-ray spectroscopy (EDS), Brunauer, Emmett, Teller (BET)/Barrett, Joyner, Halenda (BJH) analysis, inductively coupled plasma (ICP), WDX, SEM, and Vibrating Sample Magnetometry (VSM). The catalytic activity of the synthesized catalyst was evaluated in the synthesis of tetrazole and 2,3-dihydroquinazolin-4(1*H*)-one derivatives.

### Catalyst characterization

**Fig. 1:**
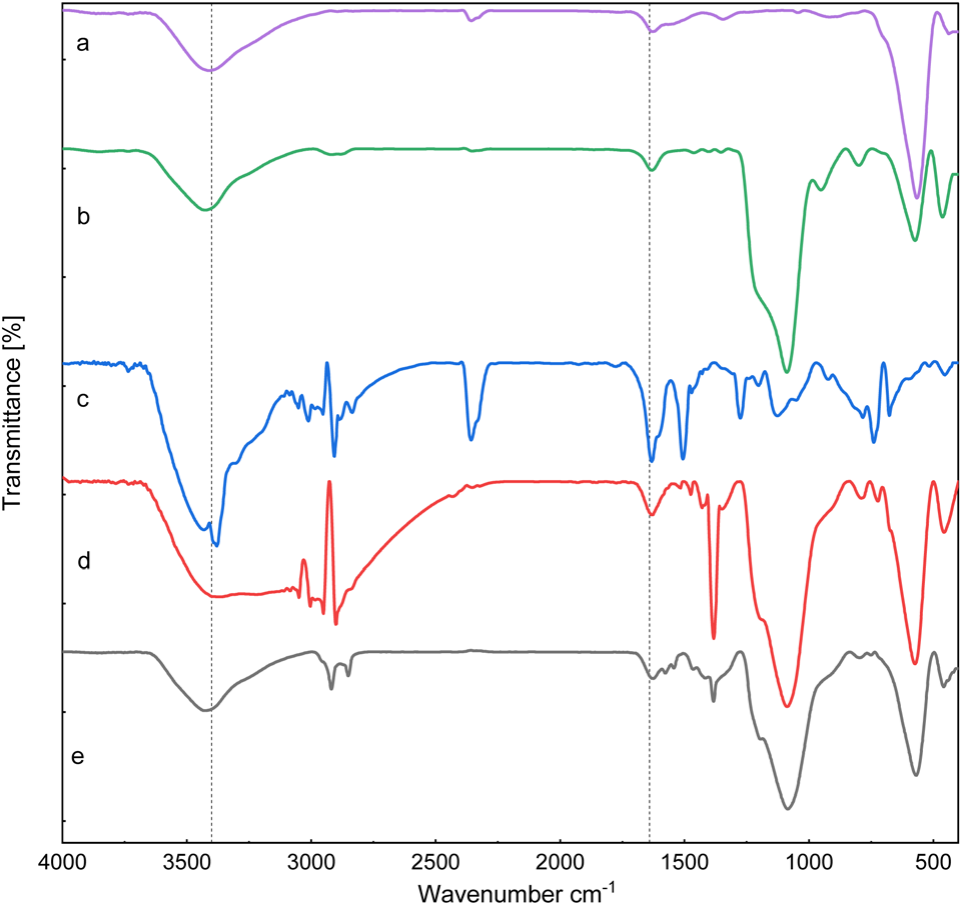
FT-IR spectra of (a) ZrFe_2_O_4_, (b) ZrFe_2_O_4_@SiO_2_, (c) GLYMO-oPD, (d) ZrFe_2_O_4_@SiO_2_@GLYMO-oPD, and (e) [ZrFe_2_O_4_@SiO_2_@GLYMO-oPD-Cu(ii)].

Fig. 1 presents the FT-IR spectra of ZrFe_2_O_4_, ZrFe_2_O_4_@SiO_2_, GLYMO-oPD, ZrFe_2_O_4_@SiO_2_@GLYMO-oPD, and the [ZrFe_2_O_4_@SiO_2_@GLYMO-oPD-Cu(ii)] complex. The FTIR spectra of ZrFe_2_O_4_ MNPs exhibited strong bands at approximately 3400 cm^−1^ and 570 cm^−1^, attributed to O–H stretching and metal–O stretching vibrations, respectively (Fig. 1a). The FTIR spectrum of ZrFe_2_O_4_@SiO_2_ (Fig. 1b) displays additional absorption bands at 1088 and 951 cm^−1^, which are related to the Si–O–Si and Si–O bending modes. These bands confirmed the successful modification of the ZrFe_2_O_4_ surface with a silica shell.^17^

The FTIR spectrum of GLYMO-oPD exhibited peaks at 2986 cm^−1^ and 3057 cm^−1^, corresponding to the stretching vibrations of sp^3^ C–H and sp^2^ C–H bonds, respectively, confirming the presence of both the GLYMO linker and *o*-phenylenediamine moiety in its structure (Fig. 1c). The absorption bands at 783 cm^−1^, 1063 cm^−1^, and 1126 cm^−1^ are assigned to the symmetric and asymmetric stretching vibrations of Si–O bonds at the end of the GLYMO-oPD molecule chain. Additionally, the peak at 3379 cm^−1^ is assigned to the stretching vibration of the NH_2_ group of *o*-phenylenediamine, while the peaks at 1471 cm^−1^ and 1506 cm^−1^ are associated with the C=C and C–N bonds, respectively. Finally, the broad peak observed between 3415 cm^−1^ and 3550 cm^−1^ are attributed to the stretching vibrations of N–H and O–H bonds. The FTIR spectrum of ZrFe_2_O_4_@SiO_2_@GLYMO-oPD (Fig. 1d), in addition to the peaks observed for ZrFe_2_O_4_@SiO_2_, exhibits characteristic peaks of GLYMO-oPD at 3404, 2952, 1626, and 1383 cm^−1^, attributed to the stretching vibrations of OH, C–H, C=C, and C–N bands, respectively. These findings indicate the successful immobilization of GLYMO-oPD onto the nanoparticles. Furthermore, the observed changes in FT-IR spectra of ZrFe_2_O_4_@SiO_2_@GLYMO-oPD-Cu(ii) in comparison to ZrFe_2_O_4_@SiO_2_@GLYMO-oPD can be attributed to coordination to copper ions (Fig. 1e).

**Fig. 2:**
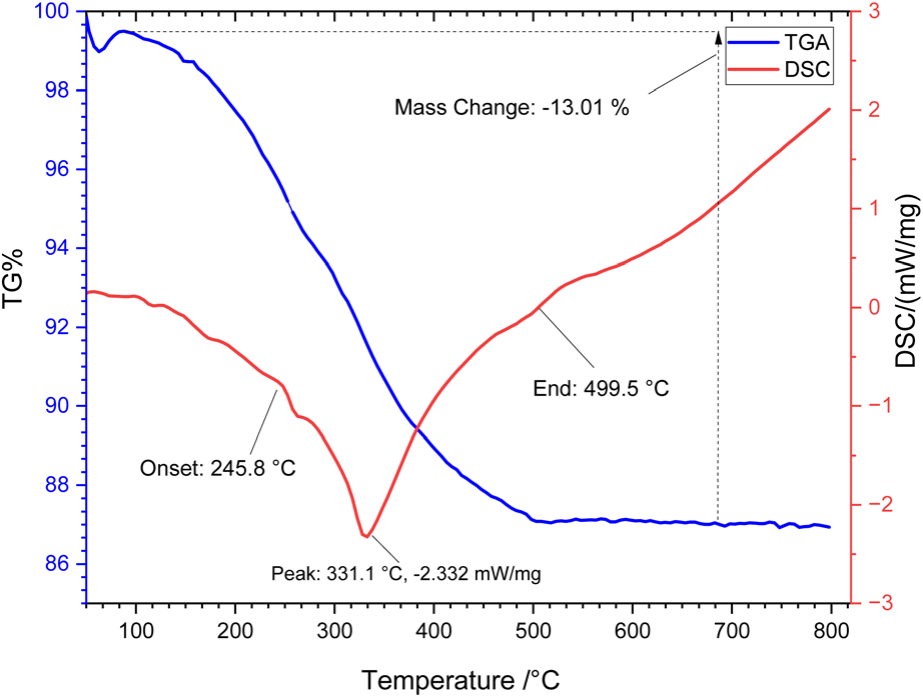
TGA and DSC curves of the [ZrFe_2_O_4_@SiO_2_@GLYMO-oPD-Cu(ii)] complex.

TGA and DSC were employed to explore the thermal behavior and organic group content of the synthesized [ZrFe_2_O_4_@SiO_2_@GLYMO-oPD-Cu(ii)] complex (Fig. 2). The TGA thermogram reveals two distinct weight loss stages. An initial weight loss of approximately 2% occurs at low temperatures (50–200 °C), corresponding to the disruption of physically adsorbed solvents and hydrogen-bonded water. The second weight loss, around 11.01%, takes place between 200 and 800 °C, related to the decomposition of the organic layers on the nanoparticle surface. Additionally, the DSC curve provides further insight into the thermal properties and phase transitions of the sample. The DSC curve exhibits an exothermic peak indicating the evaporation of surface solvents. Subsequently, a sharp exothermic peak at 331 °C is observed, likely corresponding to the decomposition of the copper complex.

**Fig. 3:**
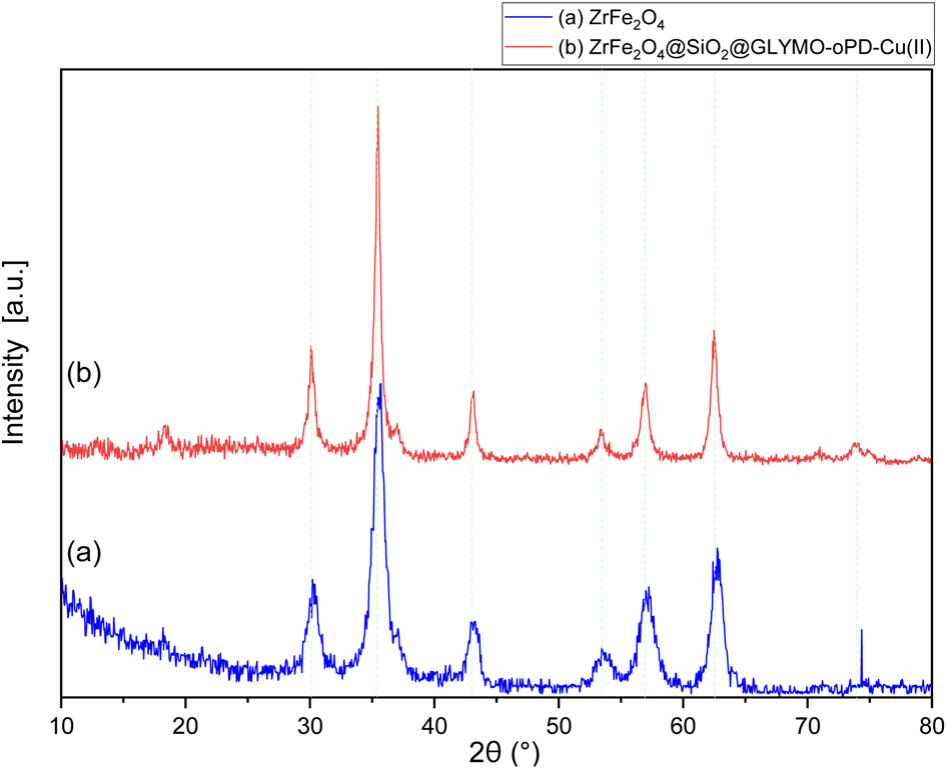
XRD patterns of ZrFe_2_O_4_ (a) and the [ZrFe_2_O_4_@SiO_2_@GLYMO-oPD-Cu(ii)] complex (b).

The XRD patterns of ZrFe_2_O_4_ and the[ZrFe_2_O_4_@SiO_2_@GLYMO-oPD-Cu(ii)] complex (Fig. 3a and b, respectively) exhibited eight distinct Bragg's diffraction peaks at specific 2*θ* values (18.35°, 30.10°, 35.46°, 43.09°, 53.41°, 56.93°, 62.48°, and 73.84°), indicative of a cubic inverse spinel structure characteristic of ZrFe_2_O_4_ MNPs (standard card JCPDS no. 01-088-0315).^22^ The good agreement between the experimental and standard XRD patterns suggests that the crystalline phase of the magnetic core remained intact after the coating process. The average crystallite size of ZrFe_2_O_4_ and the ZrFe_2_O_4_@SiO_2_@GLYMO-oPD-Cu(ii) nanocatalyst was calculated to be 19.37 and 36.31 nm, respectively, using the Debye–Scherrer formula at the peak 2*θ* = 35.46°.

**Fig. 4:**
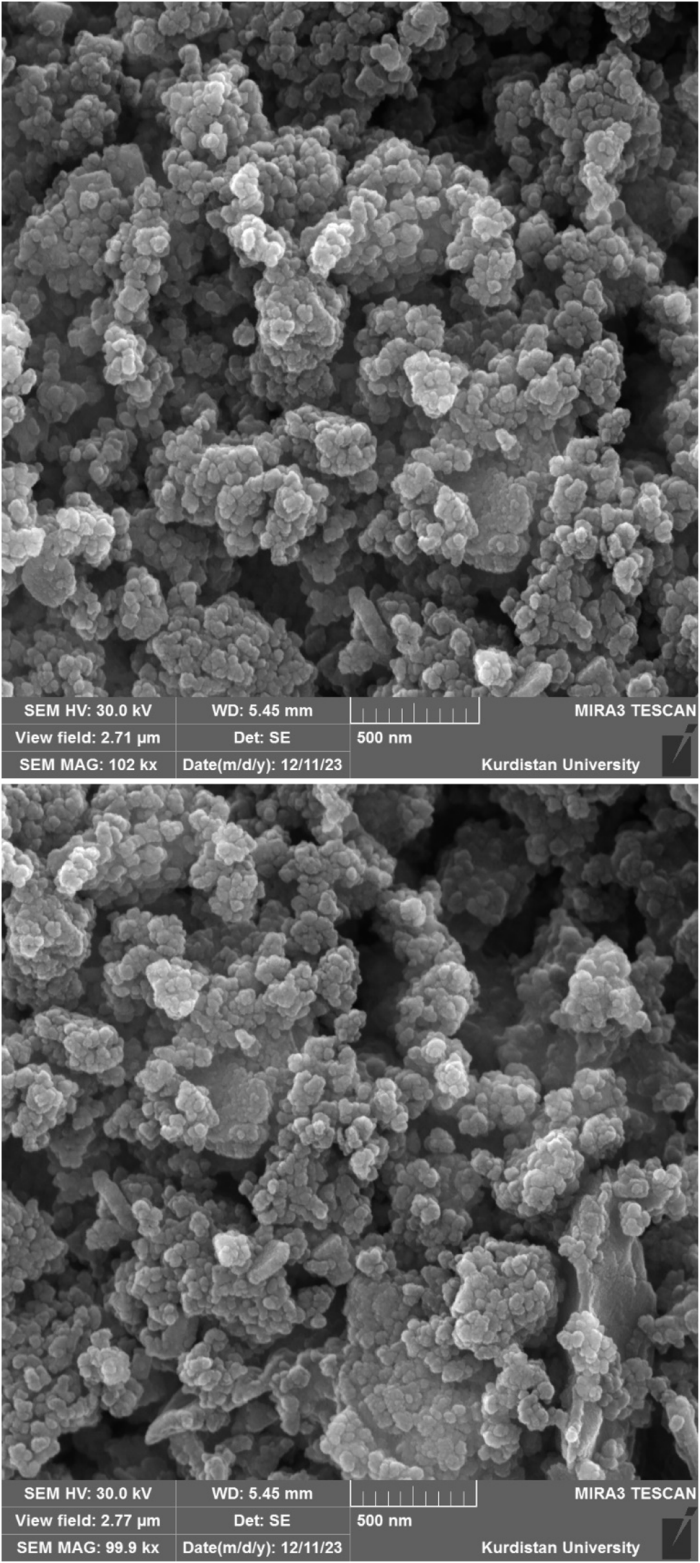
FESEM images of the [ZrFe_2_O_4_@SiO_2_@GLYMO-oPD-Cu(ii)] complex.

**Fig. 5:**
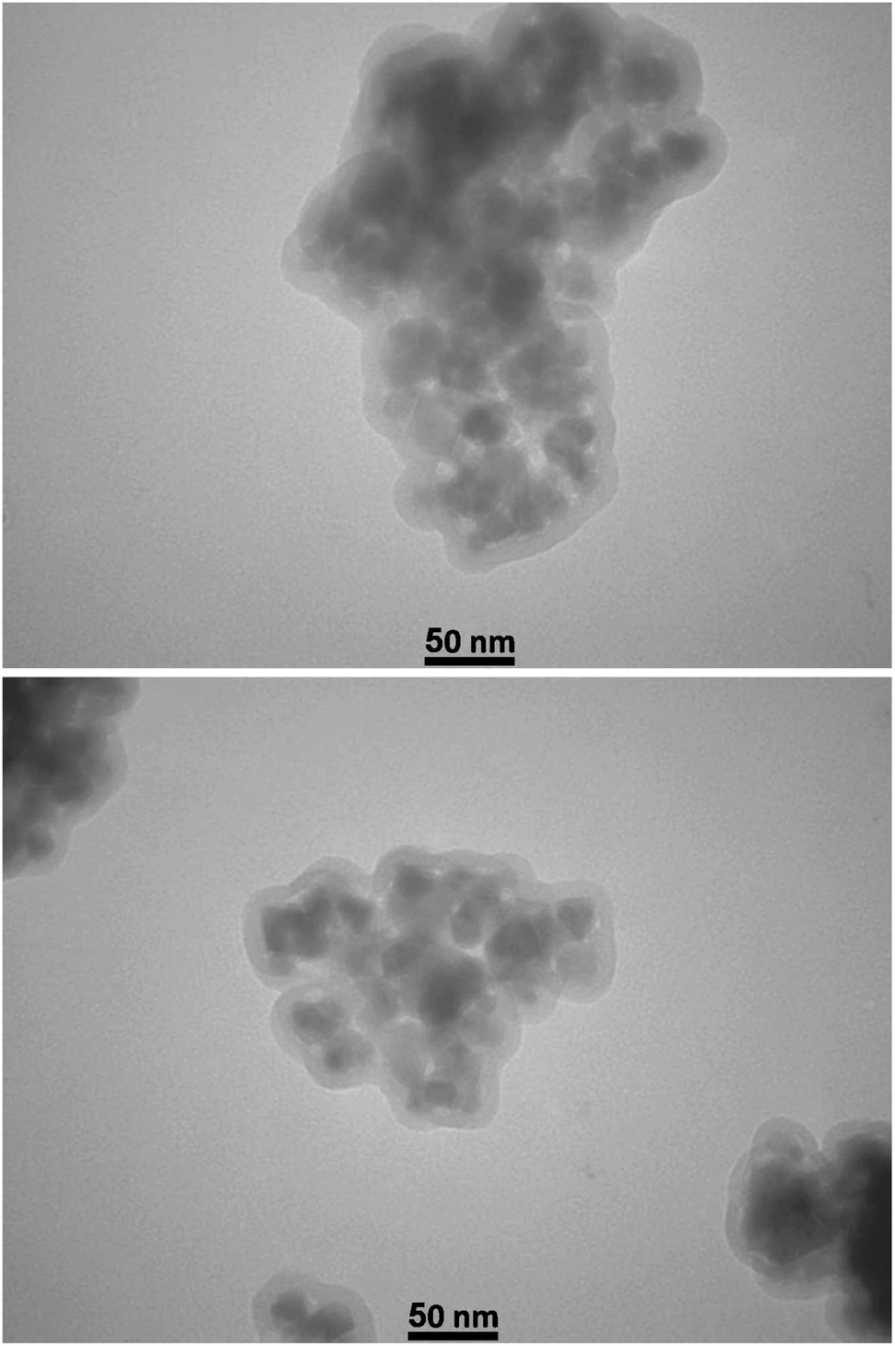
TEM images of the [ZrFe_2_O_4_@SiO_2_@GLYMO-oPD-Cu(ii)] complex.

The surface morphology of the [ZrFe_2_O_4_@SiO_2_@GLYMO-oPD-Cu(ii)] complex was investigated using FE-SEM analysis (Fig. 4). FE-SEM images revealed clusters of nanoparticles exhibiting a core–shell structure. The nanoparticles displayed a spherical morphology with a relatively uniform size distribution ranging from approximately 30 to 60 nm. The nanoparticle surfaces appeared relatively smooth, with minor surface roughness likely attributable to the presence of defects or functional groups. These observations suggest that the synthesis process effectively yielded nanoparticles within the desired size range, which could potentially enhance catalytic activity due to the increased surface-to-volume ratio characteristic of nanomaterials. Besides, the evaluation of the TEM image (Fig. 5), while confirming the accuracy of the SEM images, showed a particle size of about 10–40 nm with a spherical shape.

**Fig. 6:**
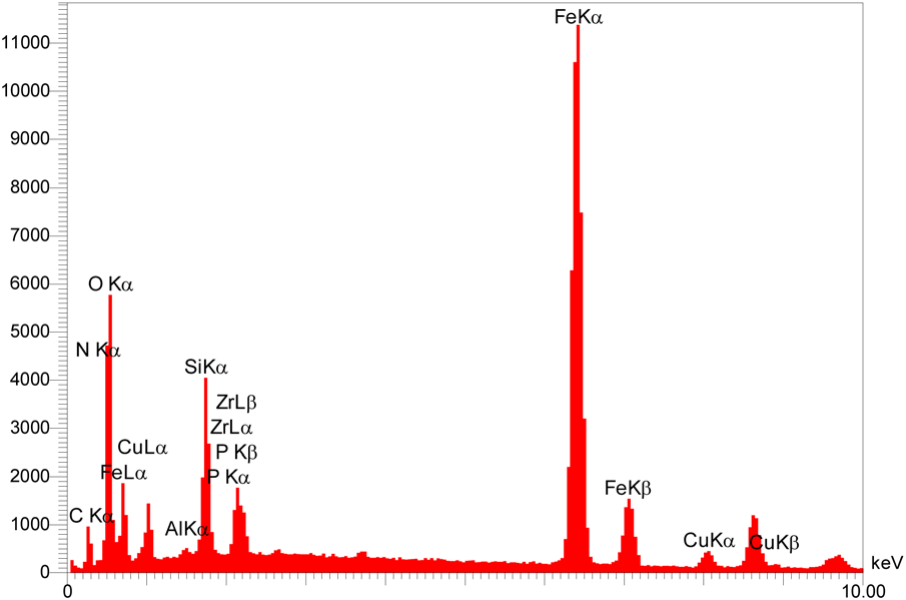
EDX spectra of the [ZrFe_2_O_4_@SiO_2_@GLYMO-oPD-Cu(ii)] complex.

The elemental composition of the ZrFe_2_O_4_@SiO_2_@GLYMO-oPD-Cu(ii) complex was investigated using EDX analysis. As depicted in Fig. 6, the EDX spectrum of the complex exhibited distinct peaks characteristic of Zr, Fe, O, Si, C, N, and Cu confirming the presence of these elements within the material. Notably, the presence of copper peaks in the EDX spectrum, coupled with their correlation with the nitrogen content, provides compelling evidence for the successful coordination of copper within the ZrFe_2_O_4_@SiO_2_@GLYMO-oPD-Cu(ii) complex. Additionally, ICP analysis confirmed the presence of copper in the prepared complex, with a copper loading of 0.44 mmol g^−1^. These quantitative data further support the successful incorporation of copper into the material.

**Fig. 7:**
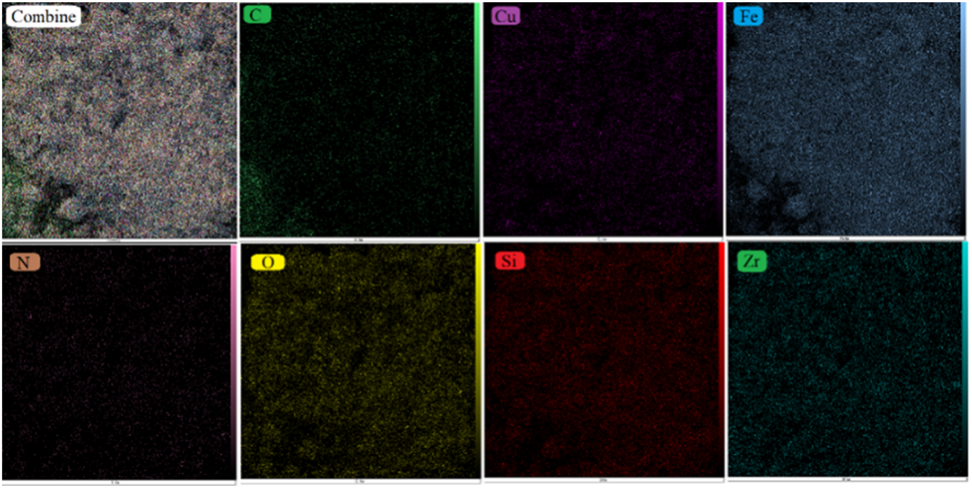
EDX elemental mapping of [ZrFe_2_O_4_@SiO_2_@GLYMO-oPD-Cu(ii)].

Elemental mapping analysis revealed the distribution of elements within the synthesized [ZrFe_2_O_4_@SiO_2_@GLYMO-oPD-Cu(ii)] complex (Fig. 7). The core region primarily consists of Zr, Fe, and O atoms, confirming the presence of ZrFe_2_O_4_ MNPs. The remaining elements, Si, C, N, and Cu, are evenly distributed across the particle surface, indicating a homogeneous coating of the coordination complex.

**Fig. 8:**
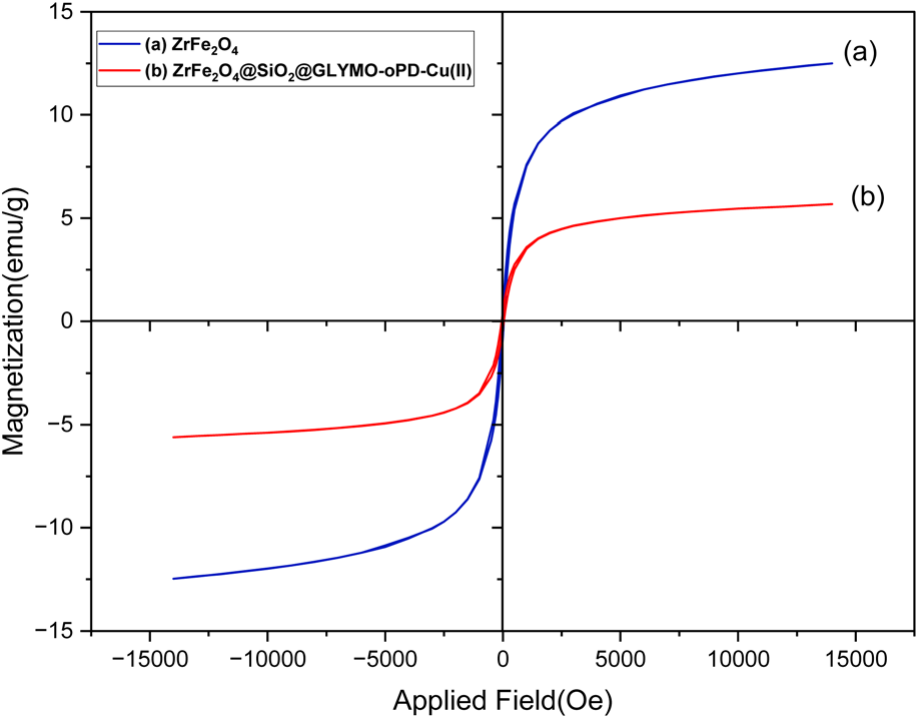
Magnetization curve of ZrFe_2_O_4_ (a) and the [ZrFe_2_O_4_@SiO_2_@GLYMO-oPD-Cu(ii)] complex (b).

VSM analysis was used to investigate the magnetic properties of ZrFe_2_O_4_ and the [ZrFe_2_O_4_@SiO_2_@GLYMO-oPD-Cu(ii)] complex (Fig. 8a and b). The complex exhibited a saturation magnetization (*M*_s_) of 5.68 emu g^−1^, which is lower than the *M*_s_ value of bare ZrFe_2_O_4_ nanoparticles of 12.50 emu g^−1^ respectively.^19^ This decrease in saturation magnetization is related to the increased size of the coordinated organometallic shells surrounding the magnetic core, which reduces the overall magnetic contribution of the ZrFe_2_O_4_ nanoparticles. Despite this, the sample can be easily demagnetized with a magnetic field, characteristic of paramagnetic materials.

**Fig. 9:**
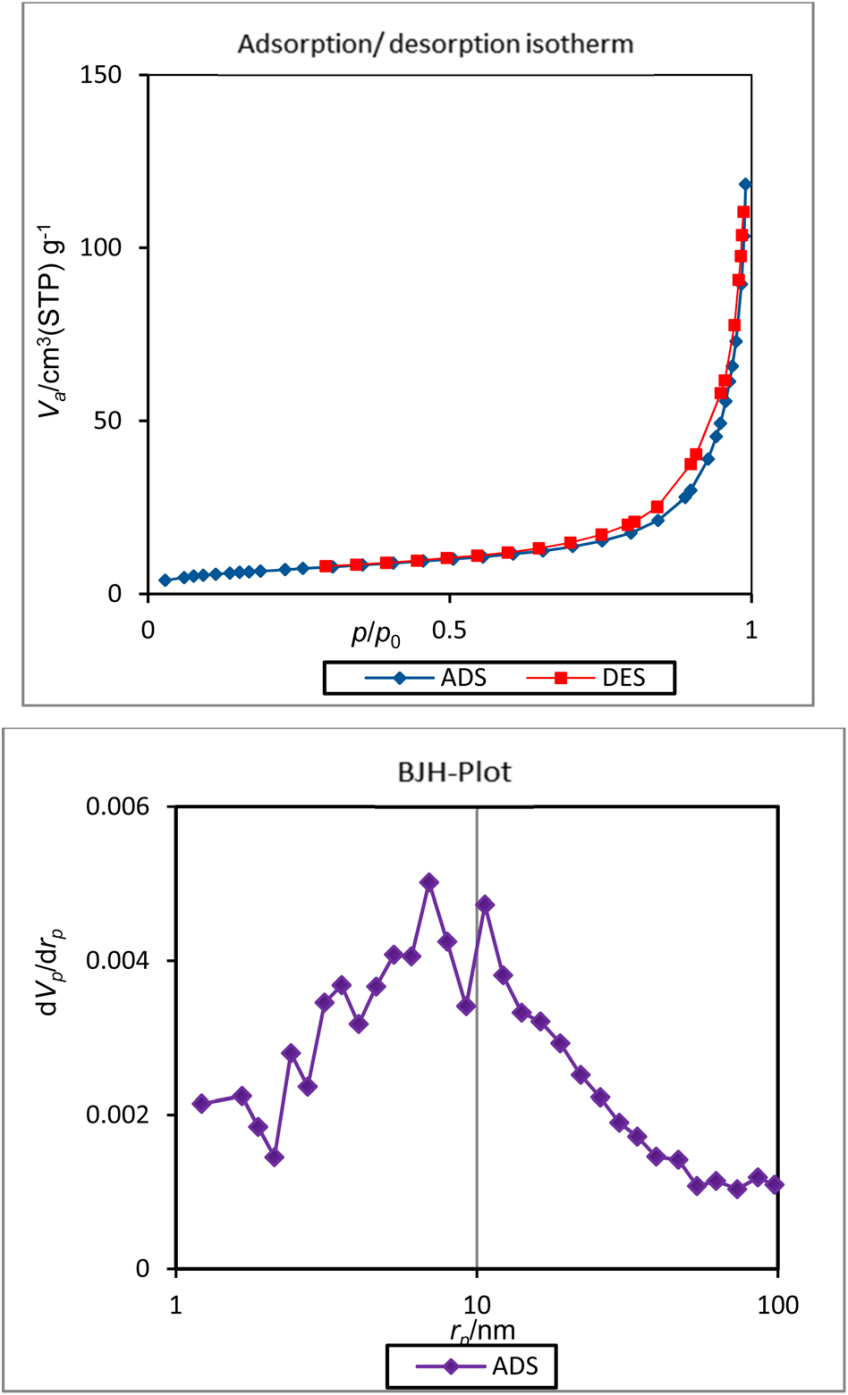
BET analysis and BJH plot of the [ZrFe_2_O_4_@SiO_2_@GLYMO-oPD-Cu(ii)] complex.

The textural properties of the [ZrFe_2_O_4_@SiO_2_@GLYMO-oPD-Cu(ii)] complex were studied by nitrogen adsorption–desorption (Fig. 9). The complex was synthesized with a mean pore diameter of about 29.6 nm, while the total pore volume and surface area were measured to be 0.18 cm^3^ g^−1^ and 24.3 m^2^ g^−1^, respectively. In addition, BJH analysis illustrates a mesopore size distribution of 2–30 nm (Fig. 9).

### Application of the catalyst

To assess the catalytic activity of the synthesized catalyst, the synthesis of two classes of nitrogen-containing heterocycles was investigated: 5-substituted 1*H*-tetrazoles and 2,3-dihydroquinazolin-4(1*H*)-ones. To optimize reaction conditions, the effects of solvent, catalyst loading, and temperature were examined for both reactions.

Initially, benzonitrile was used as a model substrate to optimize the synthesis of 5-substituted 1*H*-tetrazoles (Table 1). A comparison of catalyst-free conditions with varying catalyst loadings (10–30 mg) clearly demonstrated the catalyst's crucial role in driving the reaction (Table 1, entries 1–5). Without the catalyst, no product formation was observed, even after extended reaction times (Table 1, entries 1 and 2). Reducing the catalyst loading to 10 mg led to a 30% decrease in yield (Table 1, entry 3). Notably, using 20 mg of the catalyst provided the highest product yield, highlighting its efficiency and minimizing material waste (Table 1, entries 4 and 5). The effect of solvents on the reaction yield was evaluated by testing different solvents (Table 1, entries 4 and 6–10). At this stage, several nonpolar and polar solvents such as DMSO, H_2_O, ethanol, *n*-hexane and ethyl acetate were ineffective and resulted in incomplete conversion (Table 1, entries 6–10). The research demonstrated that the solubility of NaN_3_ plays a role as an effective parameter in the yield of the target product in polar and non-polar aprotic solvents. This reaction most likely results in a very low yield due to the insufficient solubility of NaN_3_ in the reaction medium. Among the solvents tested, PEG-400 showed a much higher yield, achieving an isolated yield of 93% (Table 1, entry 4). This behavior can be attributed to the phase transfer nature of the catalyst, which is facilitated by PEG-400. Furthermore, the complete ionization of sodium azide salt in this solvent increases the phase transfer efficiency of the catalyst. Besides, the use of PEG, as an alternative to traditional solvents, provides a less toxic and non-volatile reaction medium, an essential aspect for green chemistry. Additionally, lowering the reaction temperature resulted in decreased yields, indicating that the reaction is favored at elevated temperatures (Table 1, entries 11 and 212).

**Table 1:** Optimization of reaction conditions in the synthesis of 5-phenyl-1*H*-tetrazole

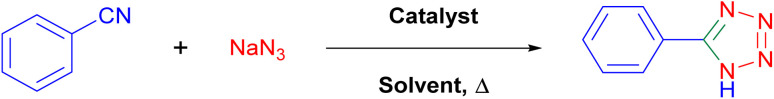						
Entry	Catalyst	Catalyst (mg)	Solvent	Temperature (°C)	Time (min)	Yield^a^^,^^b^ (%)
1	—	—	PEG-400	120	600	NR
2	ZrFe_2_O_4_	—	PEG-400	120	600	NR
3	ZrFe_2_O_4_@SiO_2_@GLYMO-oPD-Cu(ii)	10	PEG-400	120	120	30
4	ZrFe_2_O_4_@SiO_2_@GLYMO-oPD-Cu(ii)	20	PEG-400	120	40	93
5	ZrFe_2_O_4_@SiO_2_@GLYMO-oPD-Cu(ii)	30	PEG-400	120	55	80
6	ZrFe_2_O_4_@SiO_2_@GLYMO-oPD-Cu(ii)	20	EtOH	Reflux	360	N.R
7	ZrFe_2_O_4_@SiO_2_@GLYMO-oPD-Cu(ii)	20	Water	Reflux	360	N.R
8	ZrFe_2_O_4_@SiO_2_@GLYMO-oPD-Cu(ii)	20	DMSO	120	360	N.R
9	ZrFe_2_O_4_@SiO_2_@GLYMO-oPD-Cu(ii)	20	EtOAc	Reflux	360	N.R
10	ZrFe_2_O_4_@SiO_2_@GLYMO-oPD-Cu(ii)	20	*n*-Hexane	Reflux	360	N.R
11	ZrFe_2_O_4_@SiO_2_@GLYMO-oPD-Cu(ii)	20	PEG-400	100	100	75
12	ZrFe_2_O_4_@SiO_2_@GLYMO-oPD-Cu(ii)	20	PEG-400	80	150	40

a Isolated yield.

b Conditions: aryl nitrile (1.0 mmol), sodium azide (1.2 mmol), catalyst (mg) and solvent (5 mL).

After optimizing the reaction conditions, we evaluated the catalytic potential of this method for synthesizing various 5-substituted 1*H*-tetrazoles. Table 2 demonstrates that all aromatic nitrile tetrazoles containing both electron-withdrawing (NO_2_, CN, CF_3_, Cl, and Br) and electron-donating (CH_3_, OH) functional groups gave the desired tetrazoles in excellent yields. Notably, in the case of phthalonitrile, only one of the adjacent positions underwent selective transformation into the corresponding tetrazole, resulting in the formation of the tetrazole derivatives (Table 2, entry 11).

**Table 2:** Substrate scope for the synthesis of 5-phenyl-1*H*-tetrazole using the ZrFe_2_O_4_@SiO_2_@GLYMO-oPD-Cu(ii) nanocatalyst

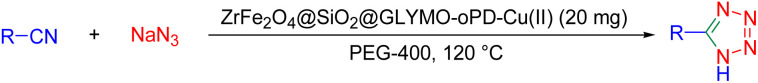				
Entry	Nitrile	Product	Time (m)	Yield^a^^,^^b^ (%)
1	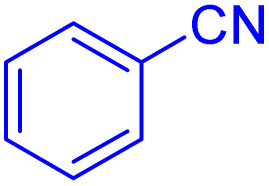	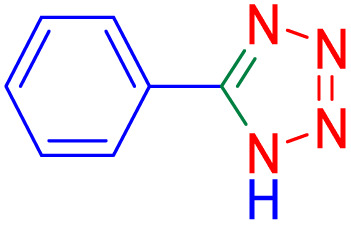	40	93
2	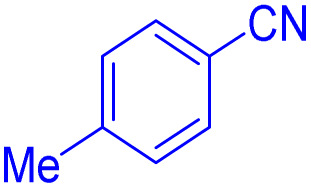	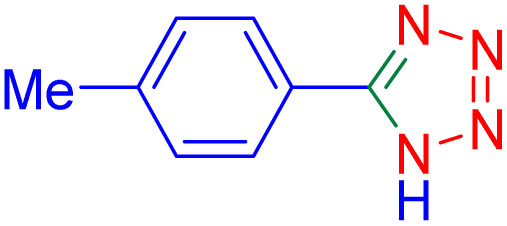	30	80
3	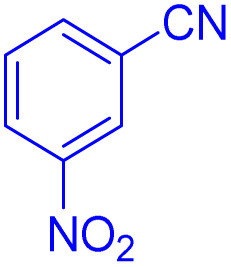	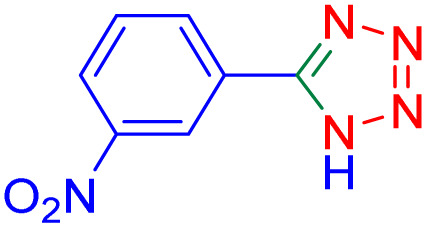	35	95
4	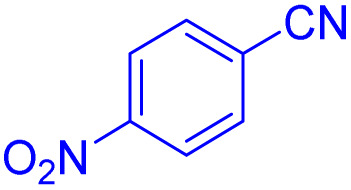	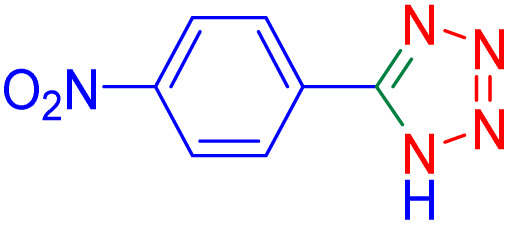	60	95
5	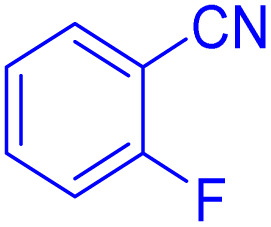	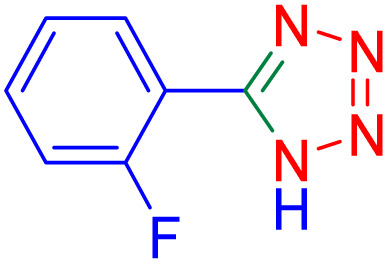	90	97
6	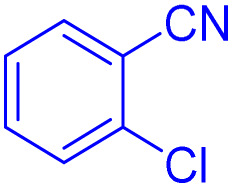	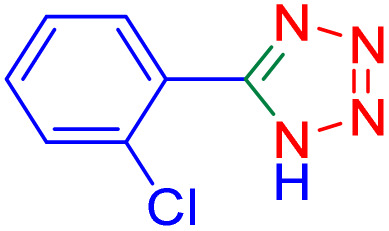	80	95
7	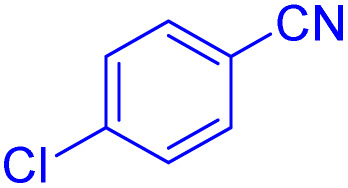	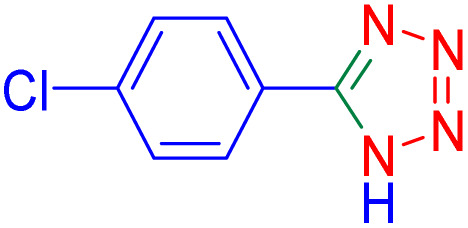	15	90
8	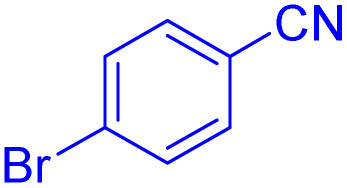	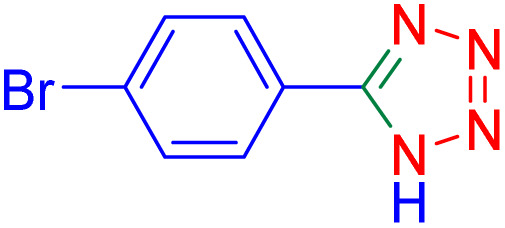	135	97
9	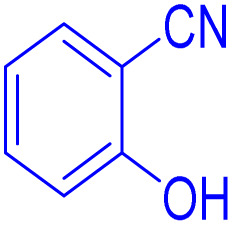	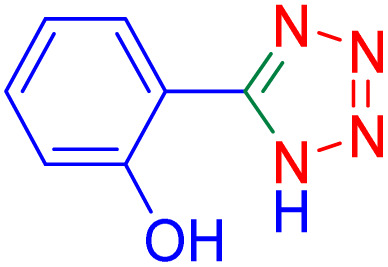	15	96
10	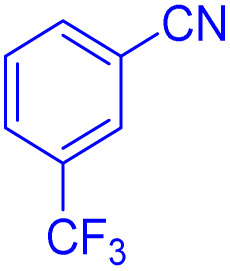	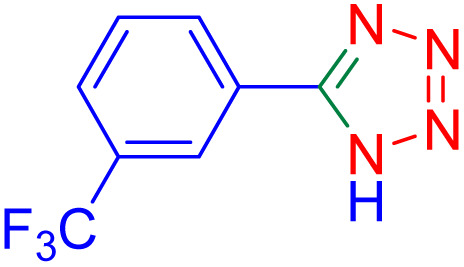	60	90
11	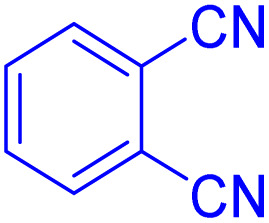	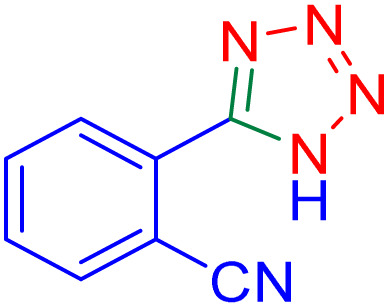	40	93

a Isolated yield.

b Conditions: aryl nitrile (1.0 mmol), sodium azide (1.2 mmol) and the ZrFe_2_O_4_@SiO_2_@GLYMO-oPD-Cu(ii) complex (20 mg) in PEG-400 (5 mL) at 120 °C.

**Scheme 2:**
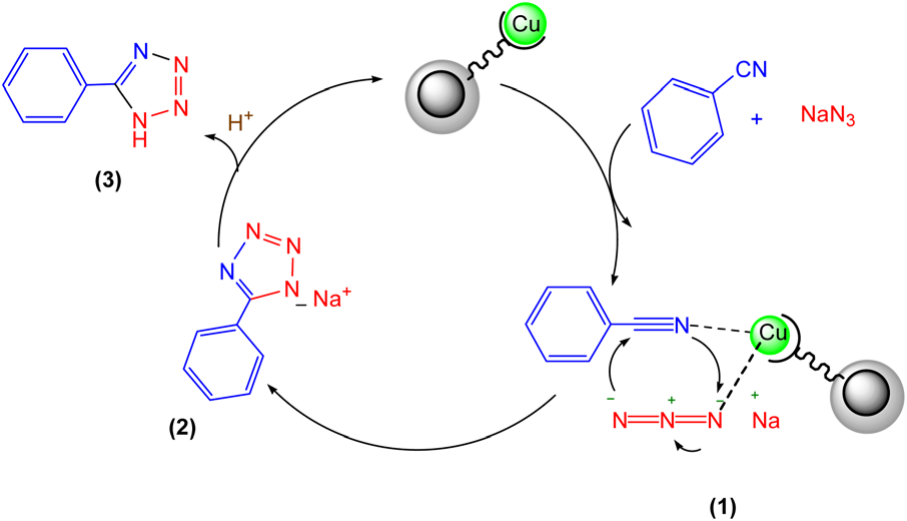
A possible mechanism for the synthesis of 5-substituted 1*H*-tetrazoles.

Based on our findings and previous literature, we propose that the most likely reaction mechanism is depicted in Scheme 2. Initially, the copper-ligand complex readily binds to nitrile, which drives the [2 + 3] cycloaddition. Subsequently, an azide molecule binds to the copper complex, allowing a nucleophilic attack to form an 18-electron complex. Hydrolysis of this complex yields the tetrazole anion, releasing the copper catalyst for further reaction cycles. Protonation of the resulting anionic intermediate upon acidification completes the reaction, producing the desired 5-aryl-1*H*-tetrazole product.^26^

The catalytic activity of [ZrFe_2_O_4_@SiO_2_@GLYMO-oPD-Cu(ii)] was further evaluated in the synthesis of 2,3-dihydroquinazolin-4(1*H*)-ones. Initially, it was investigated whether 2,3-dihydroquinazolin-4(1*H*)-one formation from 4-Cl-benzaldehyde and 2-aminobenzamide was possible using ZrFe_2_O_4_ or in the absence of a catalyst as a model substrate (Table 3, entries 1 and 2). After 12 h, the resulting yield is very poor. Therefore, catalyst amounts of about 5, 15, 25, 35 and 45 were investigated (Table 3, entries 3–7). As observed, the best amount for carrying out the reaction was 35 mg (Table 3, entry 4). By increasing the amount of catalyst to 45 mg, there was no beneficial effect on productivity and the reaction rate (Table 3, entry 3). Furthermore, to improve the product yield, the temperature effect and different solvents (EtOH, CH_2_Cl_2_, EtOAc, *n*-hexane, and DI water) were investigated (Table 3, entries 4 and 8–11). Polar protic and aprotic solvents were able to achieve moderate good yields. Among these solvents, ethanol was identified as the most efficient option, achieving 98% conversion (Table 3, entry 4). However, the use of hexane and dichloromethane for 180 min under reflux conditions did not yield satisfactory results due to their limited ability to dissolve nonpolar materials (Table 3, entries 10 and 11). Ethanol is not only greener and safer than many other solvents but also has lower toxicity and is very easy to transport and dispose of. In addition, ethanol is readily available, affordable, and can be purified. These types of reactions were also investigated at different temperatures. It was observed that the reaction at room temperature and 50 °C significantly reduced the efficiency, while at 80 °C it gave the best yield (Table 3, entries 4 and 12 and 13).

**Table 3:** Optimization of reaction conditions in the synthesis of 2,3-dihydroquinazolin-4(1*H*)-one

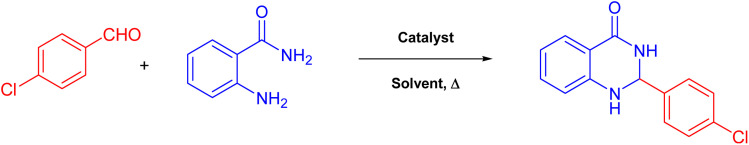						
Entry	Catalyst	Catalyst (mg)	Solvent	Temperature (°C)	Time (min)	Yield^a^^,^^b^ (%)
1	—	—	EtOH	Reflux	12 h	N.R
2	ZrFe_2_O_4_	35	EtOH	Reflux	12 h	Trace
3	[ZrFe_2_O_4_@SiO_2_@GLYMO-oPD-Cu(ii)]	45	EtOH	Reflux	55	95
4	[ZrFe_2_O_4_@SiO_2_@GLYMO-oPD-Cu(ii)]	35	EtOH	Reflux	70	98
5	[ZrFe_2_O_4_@SiO_2_@GLYMO-oPD-Cu(ii)]	25	EtOH	Reflux	90	93
6	[ZrFe_2_O_4_@SiO_2_@GLYMO-oPD-Cu(ii)]	15	EtOH	Reflux	110	78
7	[ZrFe_2_O_4_@SiO_2_@GLYMO-oPD-Cu(ii)]	5	EtOH	Reflux	180	25
8	[ZrFe_2_O_4_@SiO_2_@GLYMO-oPD-Cu(ii)]	35	DI water	Reflux	180	20
9	[ZrFe_2_O_4_@SiO_2_@GLYMO-oPD-Cu(ii)]	35	EtOAc	Reflux	180	68
10	[ZrFe_2_O_4_@SiO_2_@GLYMO-oPD-Cu(ii)]	35	CH_2_Cl_2_	Reflux	180	N.R
11	[ZrFe_2_O_4_@SiO_2_@GLYMO-oPD-Cu(ii)]	35	*n*-Hexane	Reflux	180	N.R
12	[ZrFe_2_O_4_@SiO_2_@GLYMO-oPD-Cu(ii)]	35	EtOH	50	180	68
13	[ZrFe_2_O_4_@SiO_2_@GLYMO-oPD-Cu(ii)]	35	EtOH	r.t	180	15

a Isolated yield.

b Conditions: 4-chlorobenzaldehyde (1 mmol) and 2-aminobenzamide (1 mmol), EtOH solvent (5 mL).

To further broaden the scope of this method, various aromatic aldehydes with electron-rich (CH_3_, OCH_3_, OH, and N(CH_3_)_2_) and electron-withdrawing (NO_2_, Cl, 2,4-Cl, and Br) substituents were successfully converted into the desired 2,3-dihydroquinazolin-4(1*H*)-ones in good yields and short reaction times under optimized conditions, as summarized in Table 4

**Table 4:** Substrate scope for the synthesis of 2,3-dihydroquinazolin-4(1*H*)-one derivatives using the ZrFe_2_O_4_@SiO_2_@GLYMO-oPD-Cu(ii) nanocatalyst

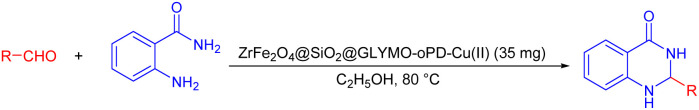				
Entry	aldehyde	Product	Time (min)	Yield^a^^,^^b^ (%)
1	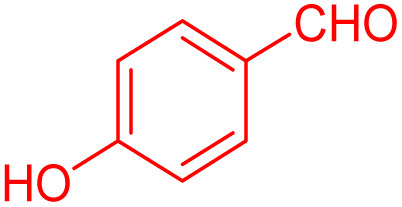	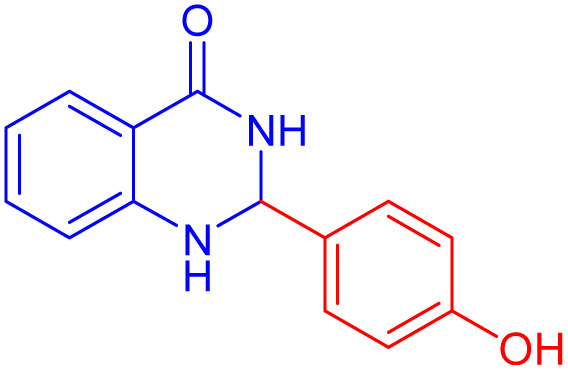	55	96
2	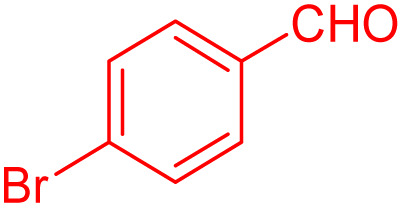	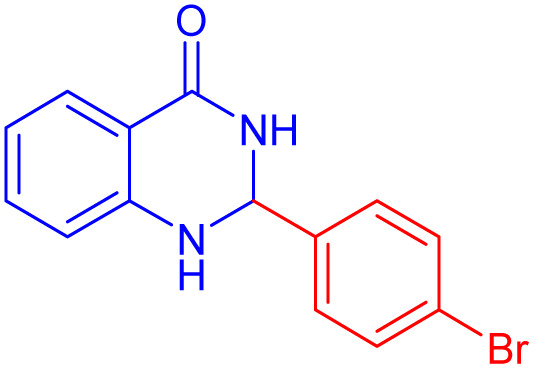	130	95
3	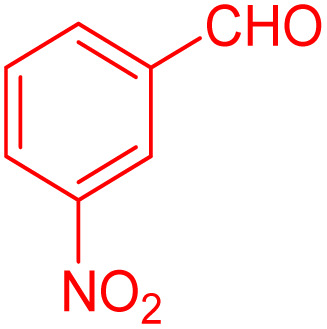	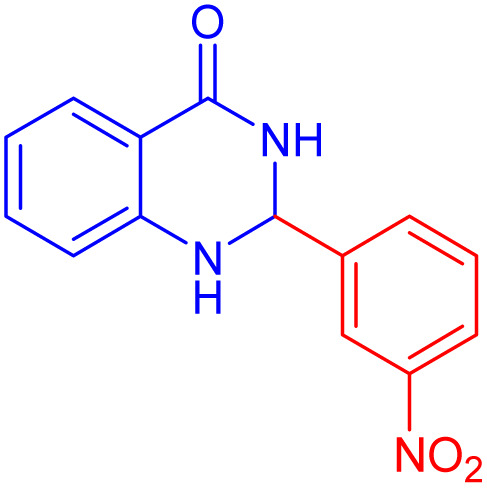	300	83
4	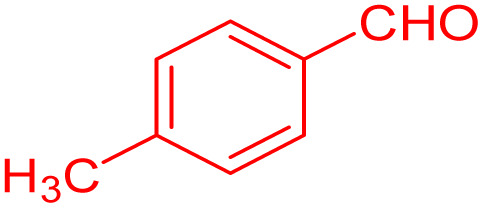	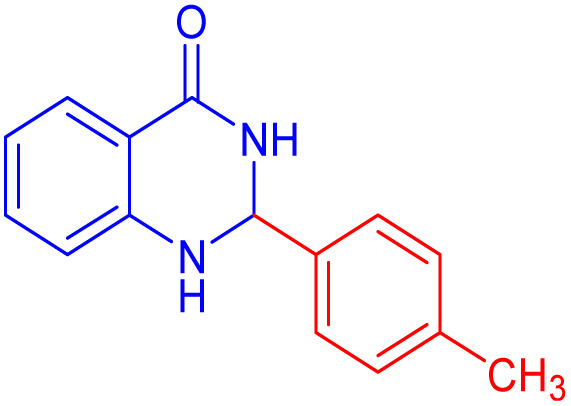	300	89
5	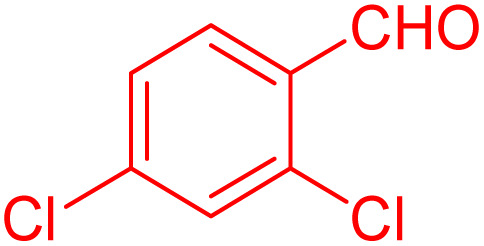	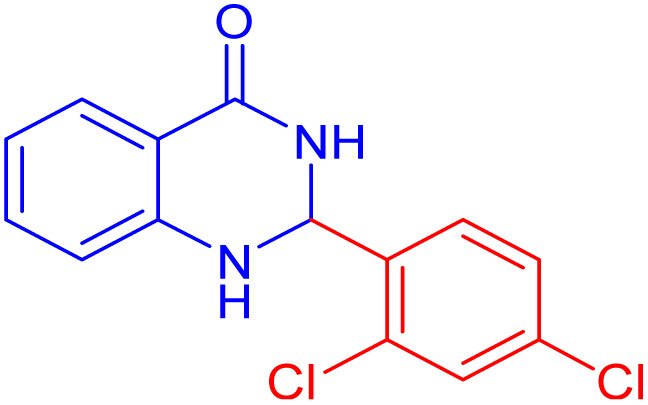	120	88
6	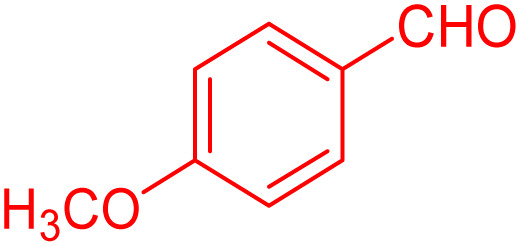	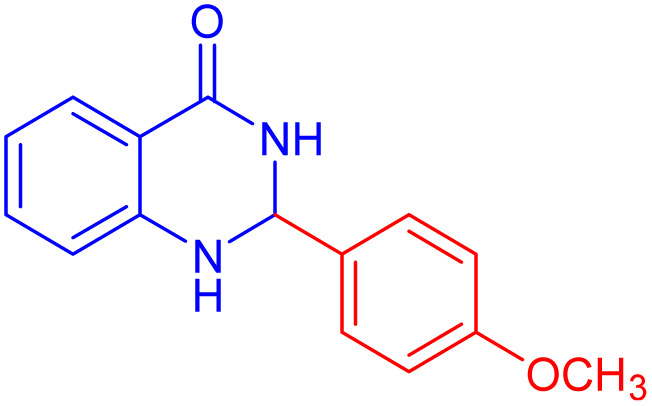	300	94
7	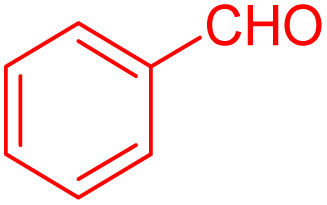	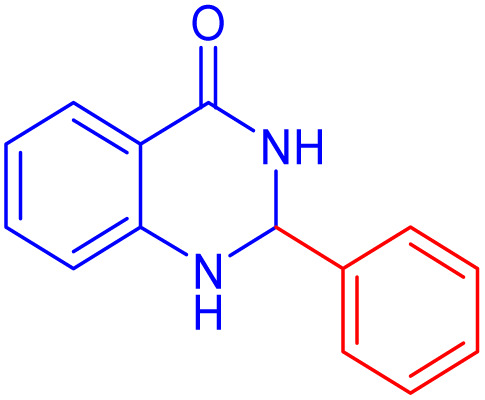	60	97
8	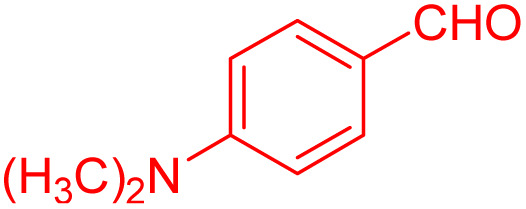	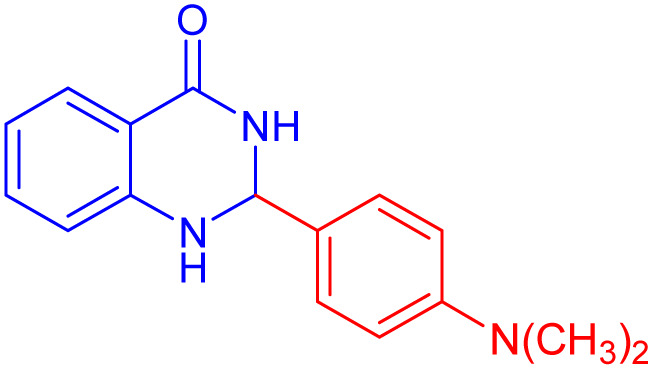	300	91
9	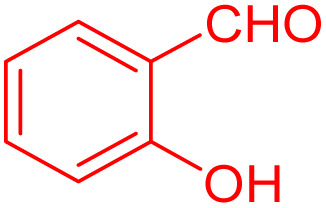	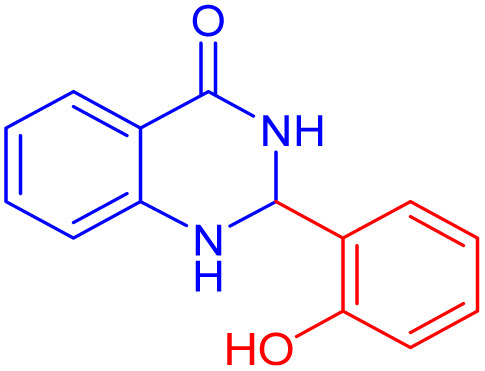	35	96
10	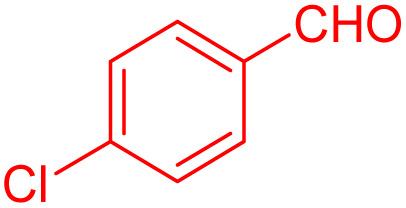	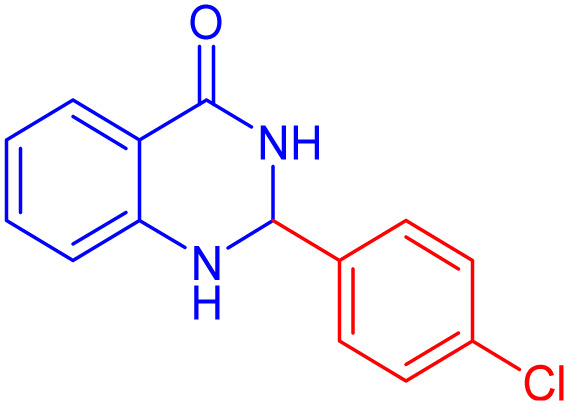	70	98

a Isolated yield.

b Conditions: 4-chlorobenzaldehyde (1 mmol) and 2-aminobenzamide (1 mmol), the [ZrFe_2_O_4_@SiO_2_@GLYMO-oPD-Cu(ii)] complex (35 mg) in EtOH solvent (5 mL).

**Scheme 3:**
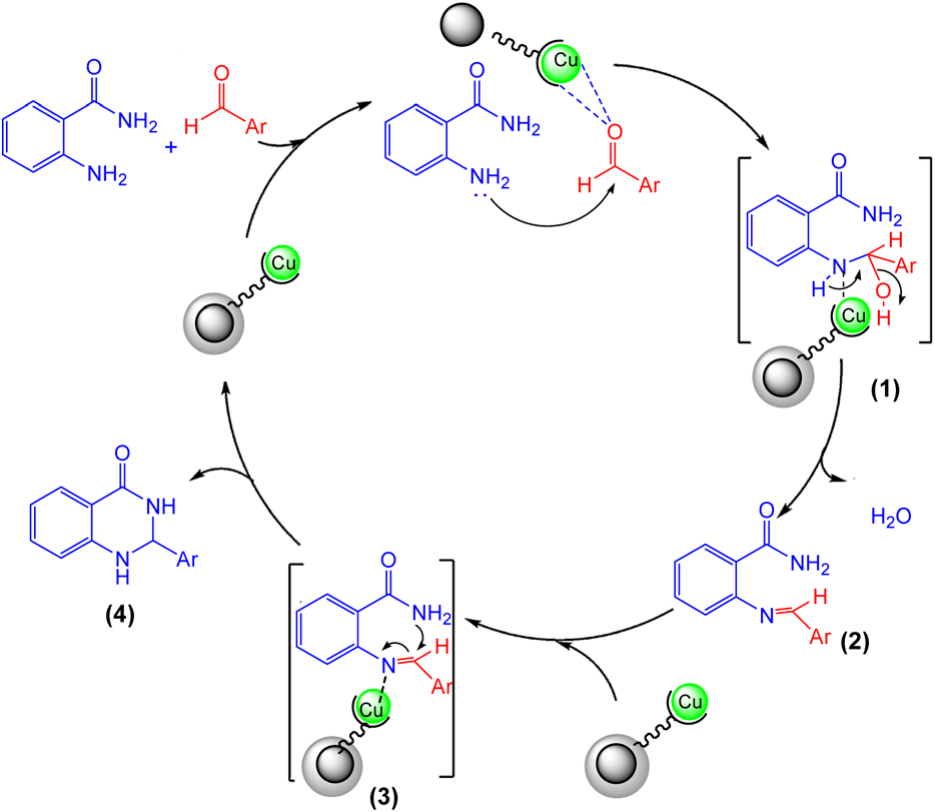
A possible mechanism for the synthesis of 2,3-dihydroquinazolin-4(1*H*)-ones.

Scheme 3 illustrates a possible mechanism for the synthesis of 2,3-dihydroquinazolin-4(1*H*)-ones catalyzed by [ZrFe_2_O_4_@SiO_2_@GLYMO-oPD-Cu(ii)]. The catalytic activity is attributed to the Lewis acidity of the copper metal center. Initially, the Cu-based nanocatalyst activates the carbonyl group of the aldehyde, facilitating nucleophilic attack by the –NH_2_ group of 2-aminobenzamide (1). Subsequently, dehydration of the intermediate, promoted by the metal center, leads to the formation of the imine intermediate (2). Finally, intramolecular cyclization occurs through the nucleophilic attack of the amide nitrogen on the activated imine group, resulting in the formation of the desired product (4).^63^

#### Catalyst recycling

**Fig. 10:**
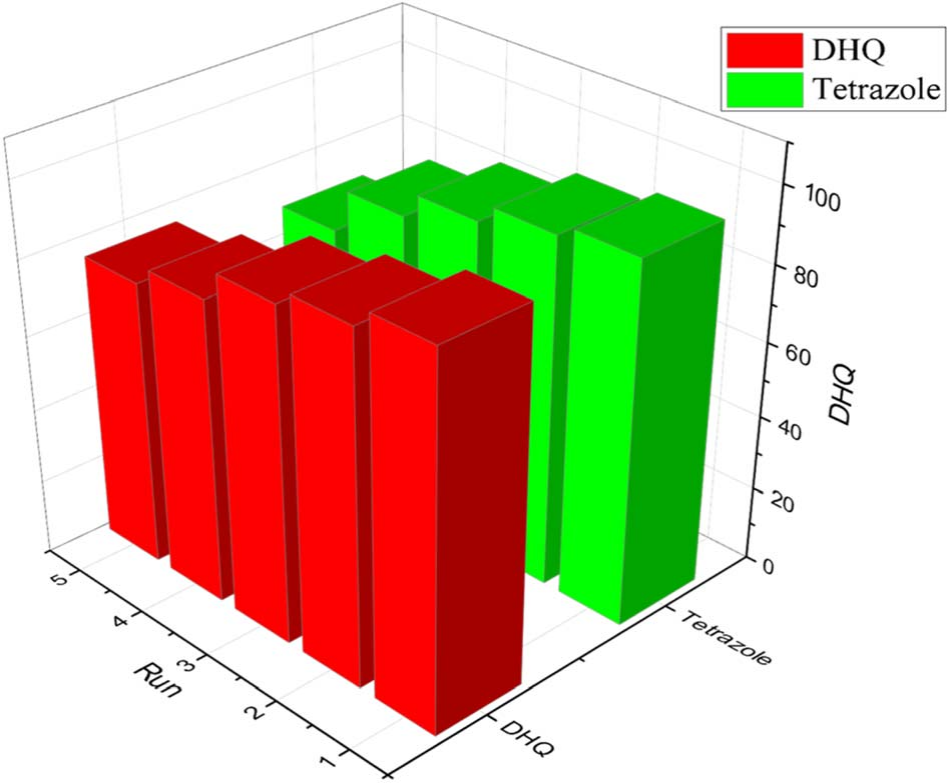
Recycling of [ZrFe_2_O_4_@SiO_2_@GLYMO-oPD-Cu(ii)]; the model reaction under optimized conditions.

The development of highly efficient and recyclable catalytic systems is a crucial aspect of green chemistry, promoting sustainable chemical synthesis and environmental protection. To assess the reusability of our novel [ZrFe_2_O_4_@SiO_2_@GLYMO-oPD-Cu(ii)] nanocatalyst, we conducted a series of model reactions. As illustrated in Fig. 10, the catalyst exhibited remarkable reusability, maintaining significant activity over at least four consecutive reaction cycles. Even after the fourth cycle, the reaction efficiency remained at a commendable 81%, demonstrating the catalyst's durability and potential for multiple uses. This impressive reusability highlights the catalyst's potential for sustainable and cost-effective chemical processes, minimizing waste generation and reducing the need for fresh catalyst production.

**Fig. 11:**
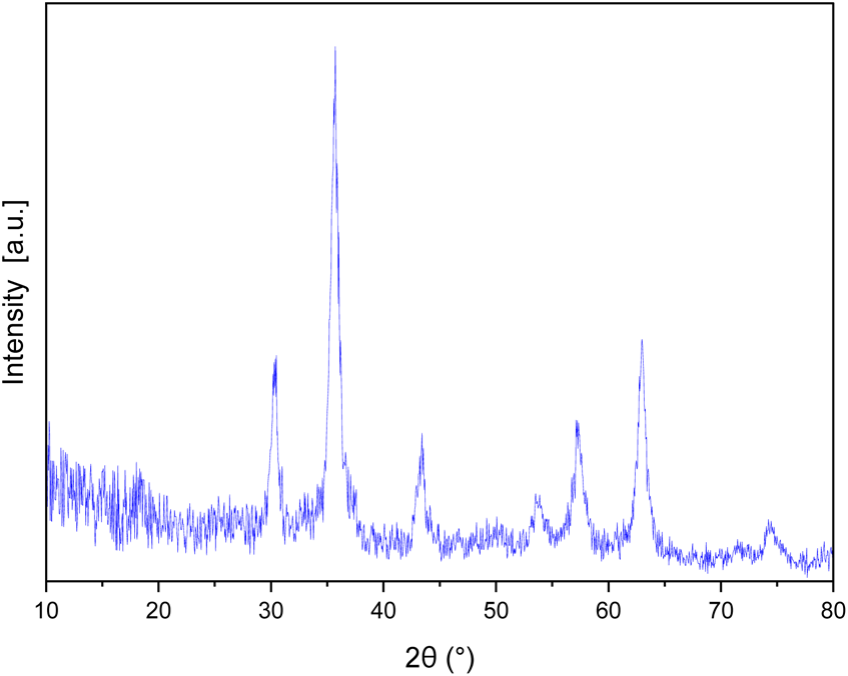
XRD pattern of the recovered nanocatalyst.

**Fig. 12:**
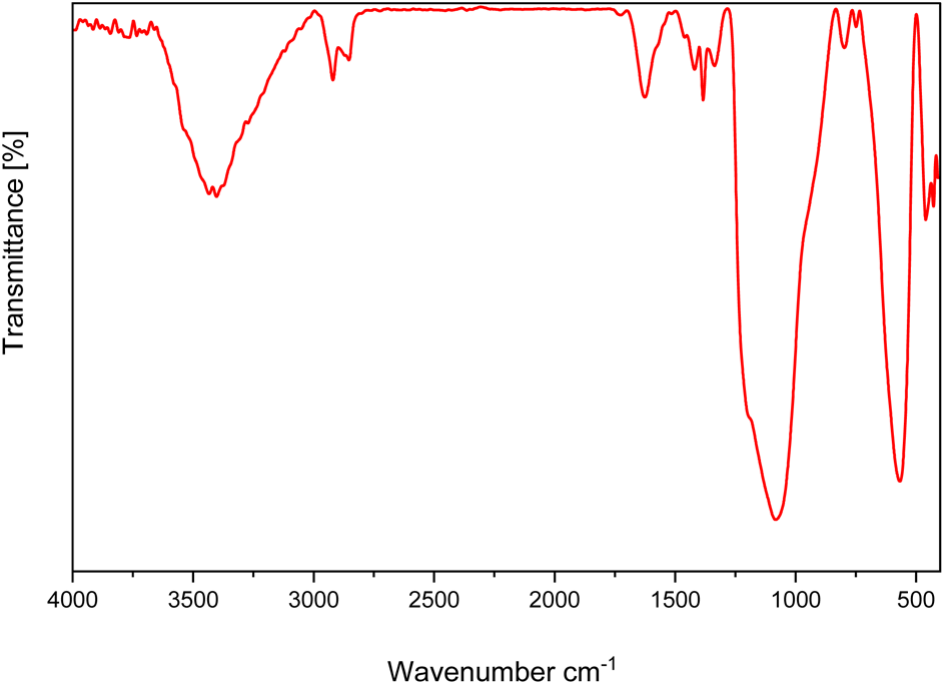
FT-IR spectrum of the recovered nanocatalyst.

The stability of the recovered catalyst was assessed over five reaction cycles using XRD and FTIR analysis. XRD analysis of the recycled nanocatalysts revealed identical diffraction patterns to those of the fresh samples, without new signals and negligible changes (Fig. 11). Similarly, the FTIR spectrum of the recycled catalyst corresponds to that of the original catalyst (Fig. 12), indicating that it preserved its structural integrity and remained active throughout the reaction-recovery-reuse process.

To assess the potential for copper leaching from the support surface during catalytic reactions, ICP analysis was conducted on the reused catalyst following multiple cycles of 2,3-dihydroquinazolin-4(1*H*)-one (Table 4, entry 10) synthesis. The results showed negligible copper leaching (0.41 mmol g^−1^), indicating that the copper complex remains firmly anchored to the support material throughout the catalytic process. This demonstrates the stability and reusability of the nanocatalyst, which is crucial for practical applications that prioritize economic and environmental sustainability.

### Comparison

Table 5 displays the synthesis of 2,3-dihydroquinazolin-4(1*H*)-one and 5-substituted 1*H*-tetrazole compounds using various catalysts in comparison with that of [ZrFe_2_O_4_@SiO_2_@GLYMO-oPD-Cu(ii)]. As indicated in this table, the [ZrFe_2_O_4_@SiO_2_@GLYMO-oPD-Cu(ii)] magnetic nanocatalyst demonstrated superior performance in terms of yield and time in these reactions. Furthermore, previous synthetic methods were usually associated with limitations, including the need to use hazardous solvents and large amounts of catalysts to achieve desired yields. These limitations not only negatively affected environmental and safety aspects but also increased operational costs. In contrast, the magnetic catalyst [ZrFe_2_O_4_@SiO_2_@GLYMO-oPD-Cu(ii)] is able to operate under milder conditions, use smaller amounts of catalyst, and use green solvents such as ethanol and PEG. As a result, this catalyst is more efficient, environmentally sustainable, and safe.

**Table 5:** Comparison of the synthesis of 5-substituted 1*H*-tetrazole [entries 1–13] and 2,3-dihydroquinazolin-4(1*H*)-one [entries 15–25] compounds in model reactions by using different catalysts

Entry	Catalyst	Time (min)	Yield (%)	Ref.
1	Fe_3_O_4_@HT@AEPH_2_-Co^II^	60	95	49
2	Fe_3_O_4_@l-lysine-Pd(0)	60	99	50
3	CoFe_2_O_4_@glycine-Yb	140	93	51
4	Fe_3_O_4_@SiO_2_–APTES–TFA	50	96	52
5	Nano-Fe_3_O_4_	270	93	53
6	Amberlyst-15	720	93	54
7	Fe_3_O_4_@MCM-41@Cu–P2C	180	95	55
8	Fe_3_O_4_@SiO_2_-aminotet-Cu(ii)	150	86	56
9	Co-(PYT)_2_@BNPs	120	98	57
10	B(C_6_F_5_)_3_	580	95	58
11	Cu(ii) immobilized on Fe_3_O_4_@ SiO_2_@l-histidine	240	95	59
12	SO_3_H@MCM-41	120	90	60
13	Fe_3_O_4_/SBA-15	180	75	61
14	ZrFe_2_O_4_@SiO_2_@GLYMO-oPD-Cu(ii)	40	93	This work
15	Fe_3_O_4_@diaza crown ether@Ni	50	95	62
17	CuCl2/Fe_3_O_4_ – TEDETA	60	95	63
18	Nylon@SO_3_H	30	97	64
19	Fe_3_O_4_@NCs-PA	60	95	65
20	Fe_3_O_4_@SiO_2_-TA-SO_3_H	45	97	66
21	Silica sulfuric acid	180	81	67
22	Fe_3_O_4_–Schiff base of Cu(ii)	210	95	68
23	Fe_3_O_4_@SiO_2_@DOPisatin-Ni(ii)	85	93	69
24	Betacyclodextrin	150	91	70
25	ZrFe_2_O_4_@SiO_2_@GLYMO-oPD-Cu(ii)	70	98	This work

## Conclusion

In conclusion, we have successfully developed a practical and efficient method for synthesizing complex copper nanocatalysts supported on magnetic zirconium ferrite. The synthesis involves a straightforward and sustainable approach, utilizing readily available and inexpensive precursors. The resulting catalyst demonstrates remarkable catalytic activity in the synthesis of biologically important heterocyclic compounds, such as tetrazoles and quinazolines. The catalyst exhibits excellent versatility, accommodating a wide range of substrates. Moreover, the copper complex remains firmly anchored to the support, minimizing leaching and ensuring the catalyst's stability and reusability. The incorporation of magnetic cores and the organometallic complex facilitates easy recovery and separation, further enhancing the catalyst's environmental friendliness and economic viability. This work provides a promising approach for the development of efficient and sustainable catalysts for organic synthesis. A recent study introduces a novel catalyst that outperforms traditional systems and promises increased selectivity, efficiency, and environmental compatibility. However, it is important to evaluate the reaction conditions to ensure scalability and real-world application. Addressing these challenges provides a balanced perspective for the industrial adoption of the catalytic process.

## Author contributions

Tara Miladi (MSc student) performed most of the practical laboratory work as part of her master's thesis. Masoomeh Norouzi (PhD) performed supervision, conceptualization, and writing, review, and editing of the manuscript draft and final version.

## Conflicts of interest

The authors declare that they have no competing interests.

## Supplementary Material

NA-007-D4NA01058B-s001

## Data Availability

The authors declare that all the data in this manuscript are available upon request.
