# Supplementary material for: Copper(ii) complex-decorated ZrFe2O4 nanoparticles as a recyclable magnetic nanocatalyst for synthesis of N-containing heterocycles
Source: Nanoscale Adv. 2025 May 12;7(13):4104–16. doi: 10.1039/d4na01058b (PMC12108969; doi:10.1039/d4na01058b)
Supplement: NA-007-D4NA01058B-s001 [file NA-007-D4NA01058B-s001.pdf]

Supplementary Information (SI) for Nanoscale Advances.
This journal is © The Royal Society of Chemistry 2025

Copper (II) complex-Decorated $ZrFe_2O_4$ Nanoparticles as Recyclable Magnetic Nanocatalyst for Synthesis of *N*-containing heterocycles

Tara Miladi [a], Masoomeh Norouzi [a,*]

[a] Department of Chemistry, Faculty of Science, Ilam University, P.O. Box 69315516, Ilam, Iran.

*Corresponding Author E-mail: m.norozi@ilam.ac.ir

Figure S1. ^{1}HNMR spectrum of 5-(4-Chloro-phenyl)-1H-tetrazole

Figure S2. ^{1}HNMR spectrum of 5-p-Tolyl-1H-tetrazole

Figure S3. ^{1}HNMR spectrum of 5-(3-Trifluoromethyl-phenyl)-1H-tetrazole:

Figure S4. ^{1}HNMR spectrum of 5-(2-Fluoro-phenyl)-1H-tetrazole

Figure S5. ^{1}HNMR spectrum of 5-(4-Bromo-phenyl)-1H-tetrazole

Figure S6. ^{1}HNMR spectrum of 5-Phenyltetrazole

Figure S7. ^{1}HNMR spectrum of 2-(1H-Tetrazol-5-yl)-benzonitrile

Figure S8. ^{1}HNMR spectrum of 2-(4-Bromo-phenyl)-2,3-dihydro-1H-quinazolin-4-one

Figure S9. ^{1}HNMR spectrum of 2-(4-Chloro-phenyl)-2,3-dihydro-1H-quinazolin-4-one

Figure S10. ^{1}HNMR spectrum of 2-Phenyl-2,3-dihydro-1H-quinazolin-4-one

NMR Data

5-(4-Chloro-phenyl)-1H-tetrazole:

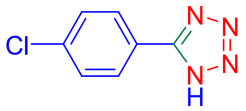

^{1}H NMR (250 MHz, DMSO-d_6) δ: 1693 (s, 1H, NH), 8.05-8.02 (d, J = 8 Hz, 2H, CHAr), 7.69-7.66 (d, J = 8 Hz, 2H, CHAr**).**

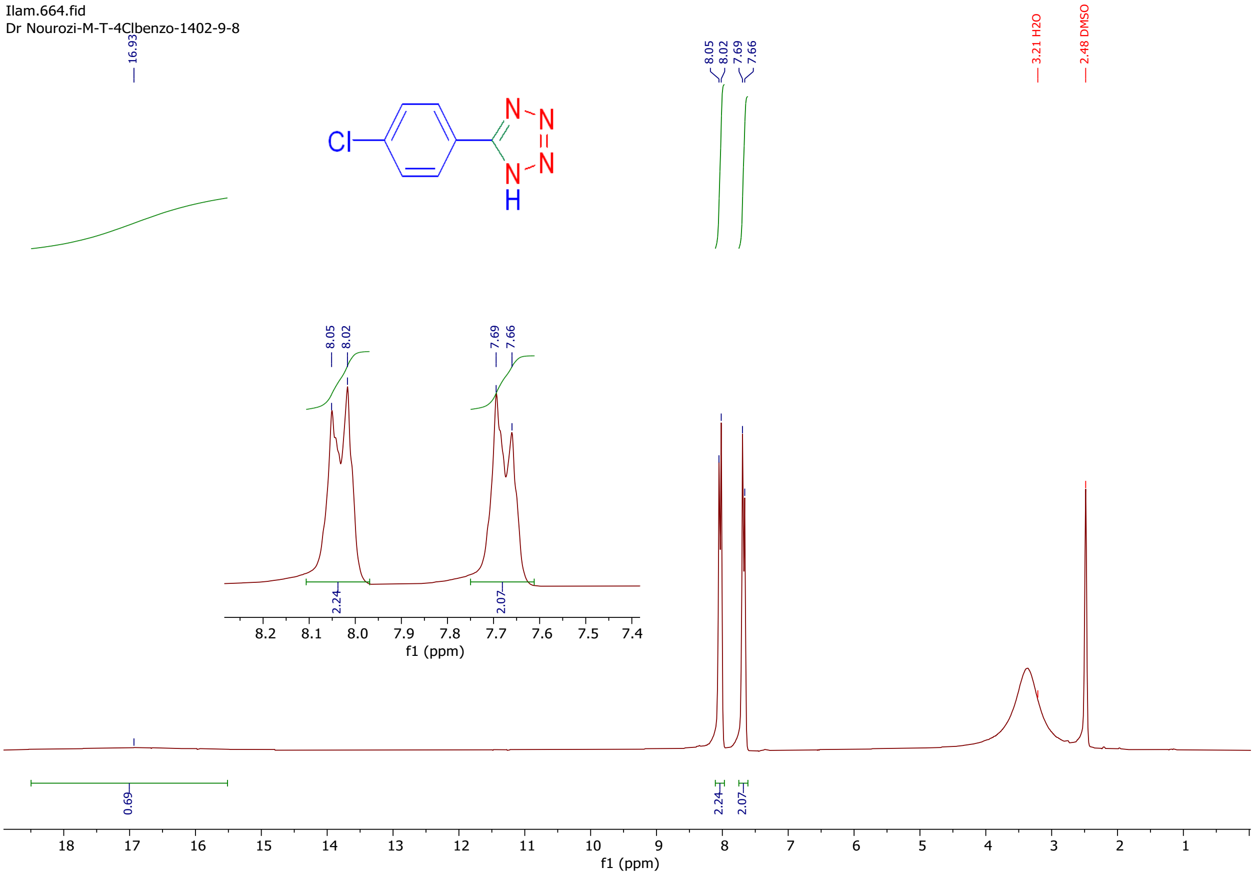

Figure S1. ^{1}HNMR spectrum of 5-(4-Chloro-phenyl)-1H-tetrazole

5-p-Tolyl-1H-tetrazole:

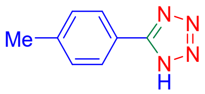

^{1}H NMR (250 MHz, DMSO-d$_6$) δ: 16.74 (s, 1H, NH), 7.93-7.89 (d, *J* = 8 Hz, 2H, CHAr), 7.41-7.38 (d, *J* = 8 Hz, 2H, CHAr), 2.33 (s, 3H, CH_3).

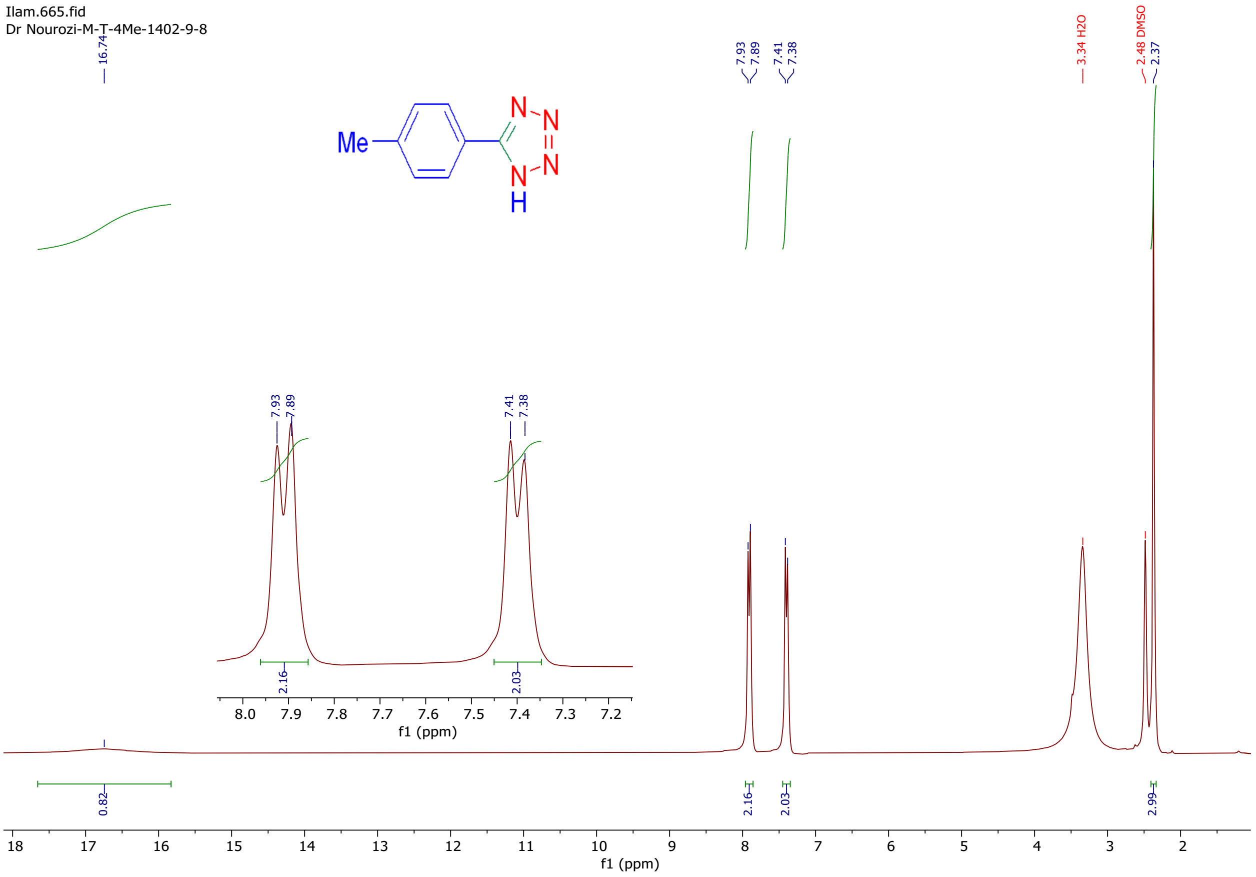

Figure S2. ^{1}HNMR spectrum of 5-p-Tolyl-1H-tetrazole

5-(3-Trifluoromethyl-phenyl)-1H-tetrazole:

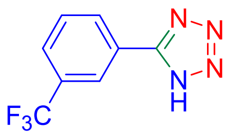

^{1}H NMR (250 MHz, DMSO-d$_6$) δ: 8.07 (s, 2H, CHAr), 7.95-7.88 (t, *J* = 7.9 Hz, 1H, CHAr), 7.80-7.73 (t, *J* = 7.9 Hz, 1H, CHAr)

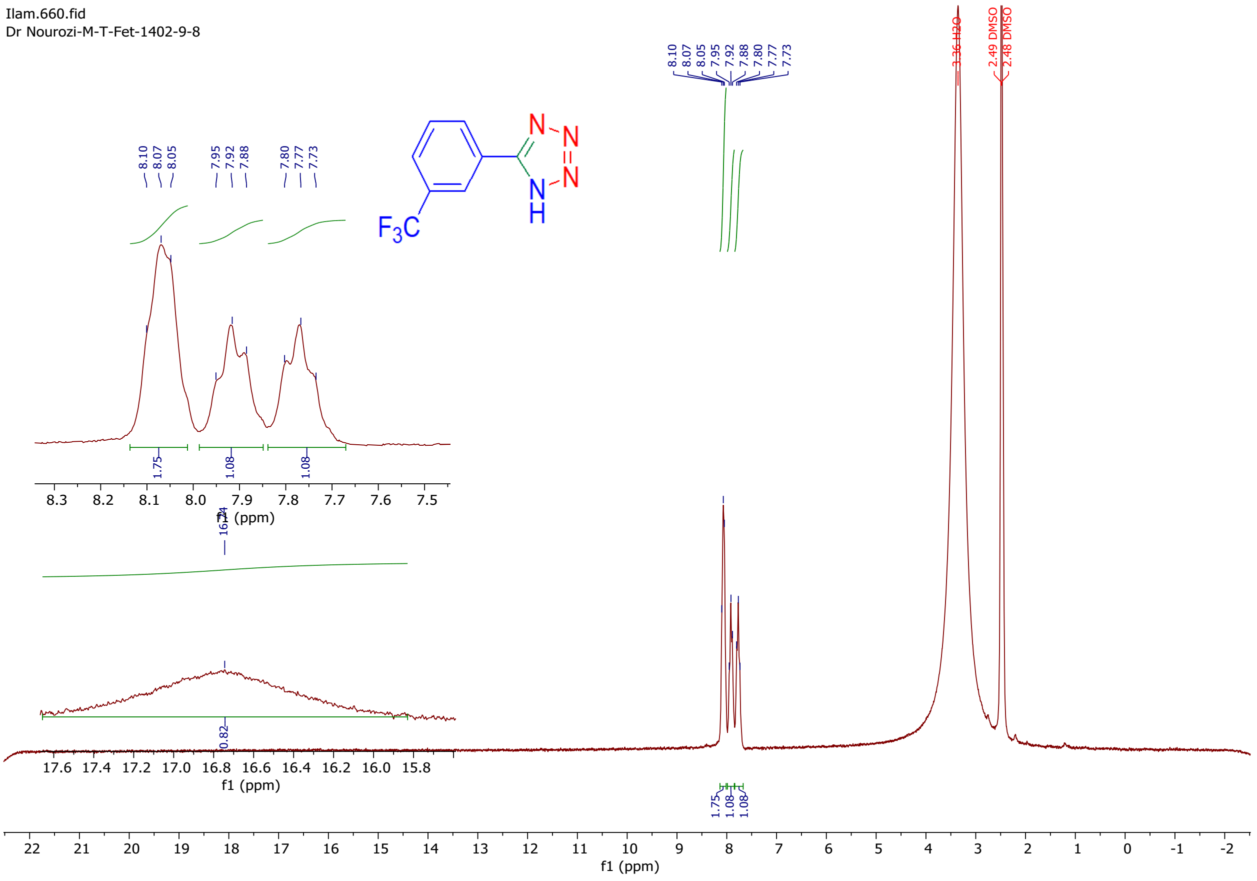

Figure S3. ^{1}HNMR spectrum of 5-(3-Trifluoromethyl-phenyl)-1H-tetrazole:

5-(2-Fluoro-phenyl)-1H-tetrazole:

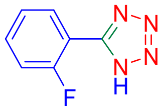

^{1}H NMR (250 MHz, DMSO-d_6) δ: 17.07 (b, 1H, N-H), 8.34 (s, 1H, CHAr), 7.97-7.93 (d, J = 8 Hz, 1H, CHAr), 7.87-7.81 (m, 2H, CHAr)

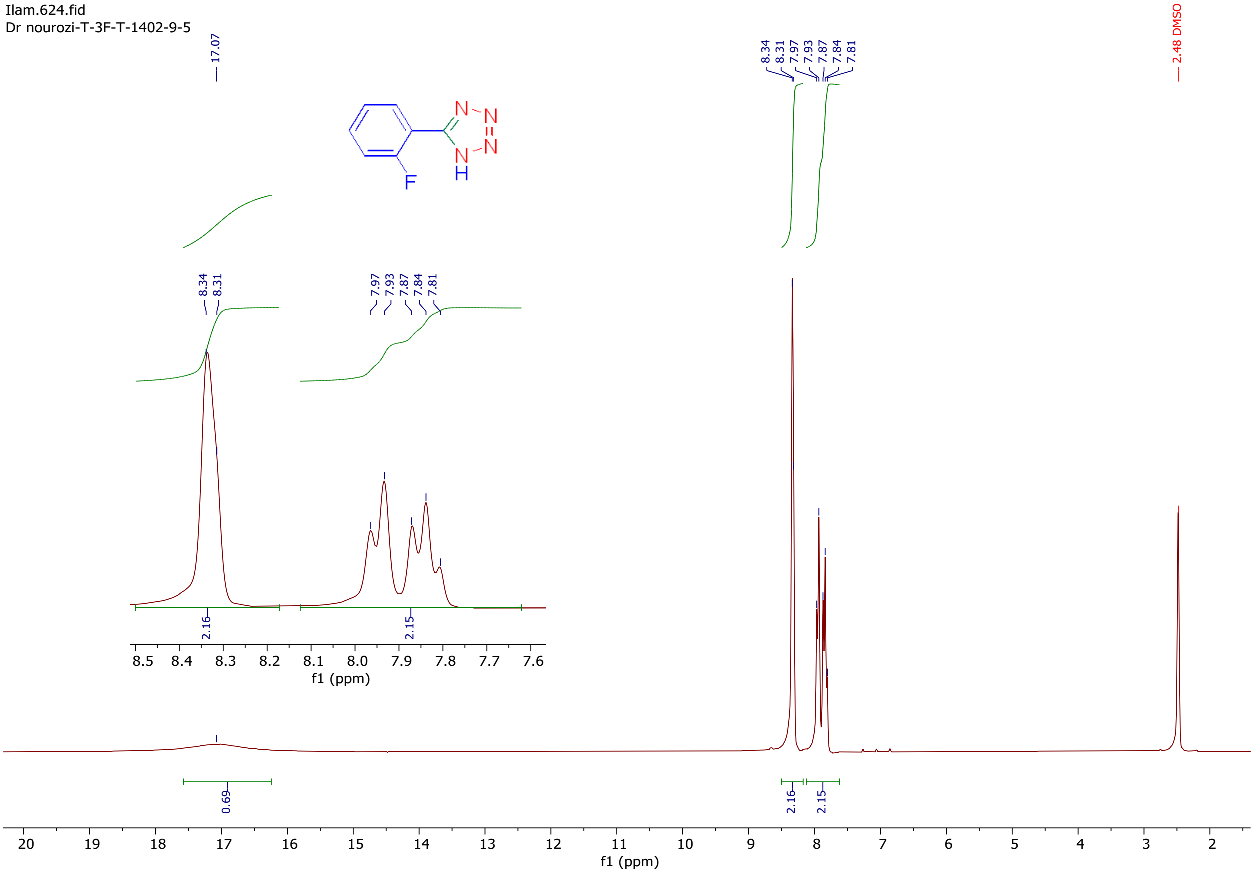

Figure S4. ^{1}HNMR spectrum of 5-(2-Fluoro-phenyl)-1H-tetrazole

5-(4-Bromo-phenyl)-1H-tetrazole:

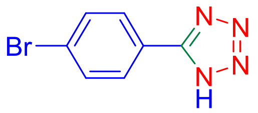

^{1}H NMR (250 MHz, DMSO-d$_6$) δ: 16.87 (b, 1H, N-H), 7.97-7.94 (d, J = 8 Hz, 2H, CHAr), 7.83-7.80 (d, J = 8 Hz, 2H, CHAr).

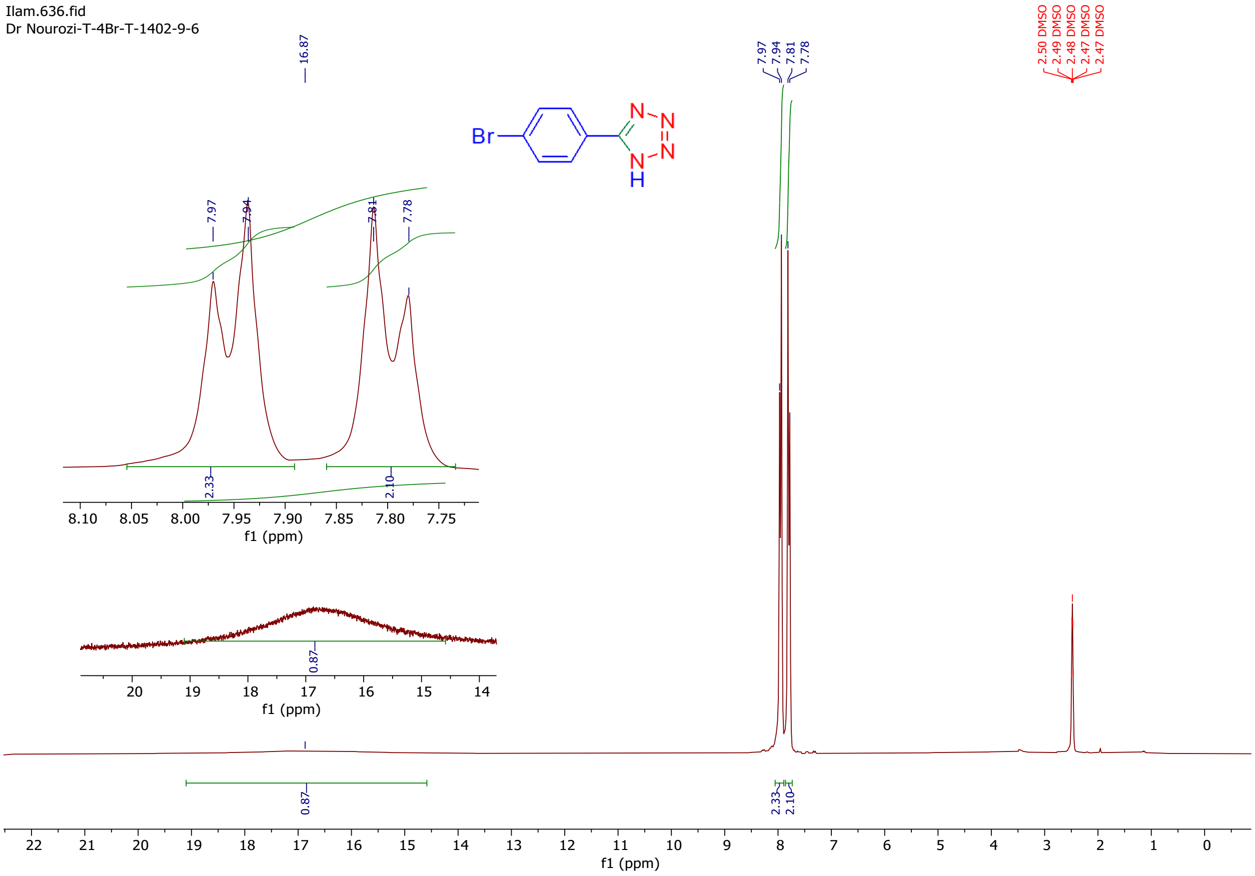

Figure S5. ^{1}HNMR spectrum of 5-(4-Bromo-phenyl)-1H-tetrazole

5-Phenyltetrazole:

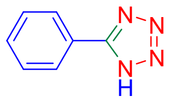

^{1}H NMR (250 MHz, DMSO-d_6) δ: 16.83 (b, 1H, N-H), 8.03-8.02 (d, *J* = 8 Hz, 2H, CHAr), 7.60 (t, *J* = 7.9 Hz, 3H, CHAr).

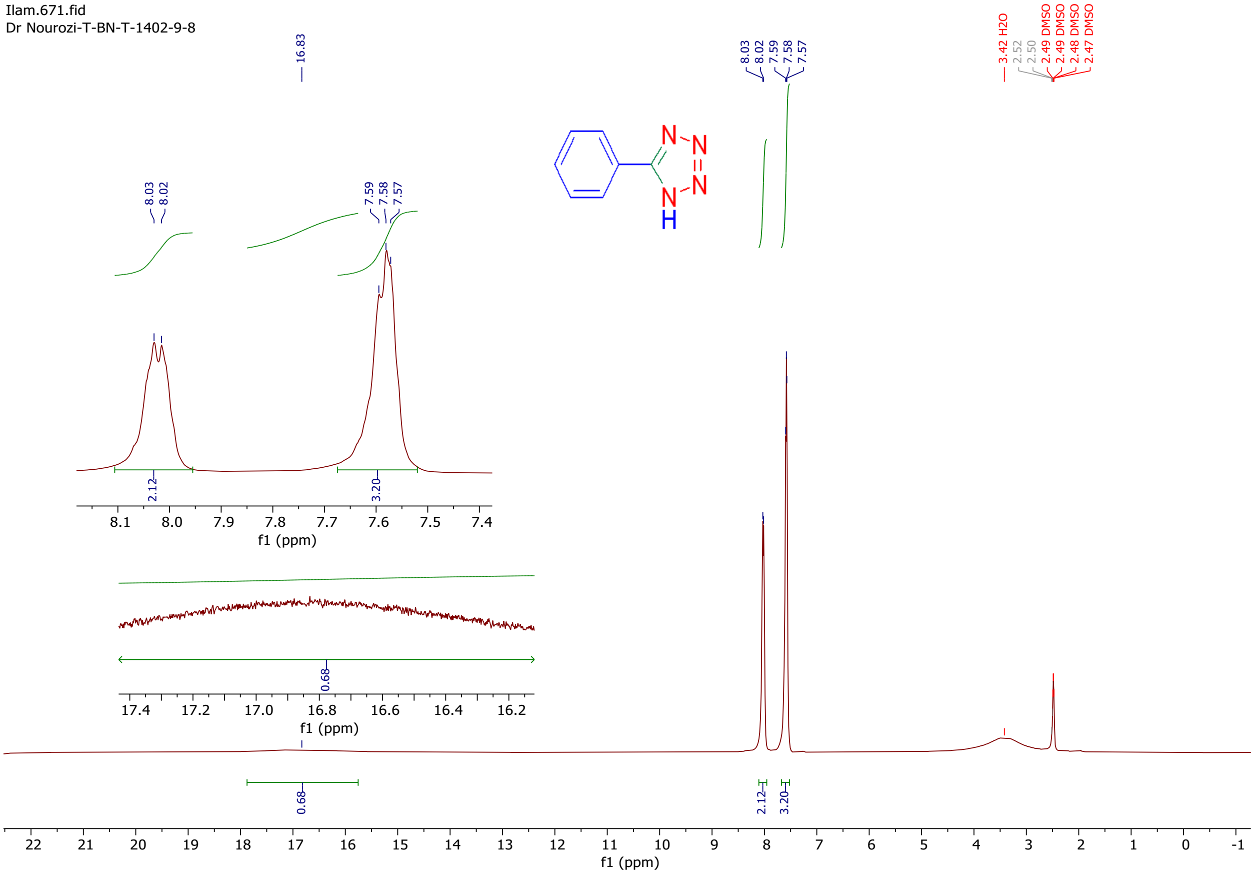

Figure S6. ^{1}HNMR spectrum of 5-Phenyltetrazole

2-(1H-Tetrazol-5-yl)-benzonitrile:

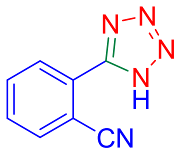

^{1}H NMR (250 MHz, DMSO-d_6) δ: 16.93 (b, 1H, N-H), 7.80-7.75 (s, 2H, CHAr), 7.94-7.88 (d, *J* = 8 Hz, 1H, CHAr), 7.79-7.76 (m, 1H, CHAr).

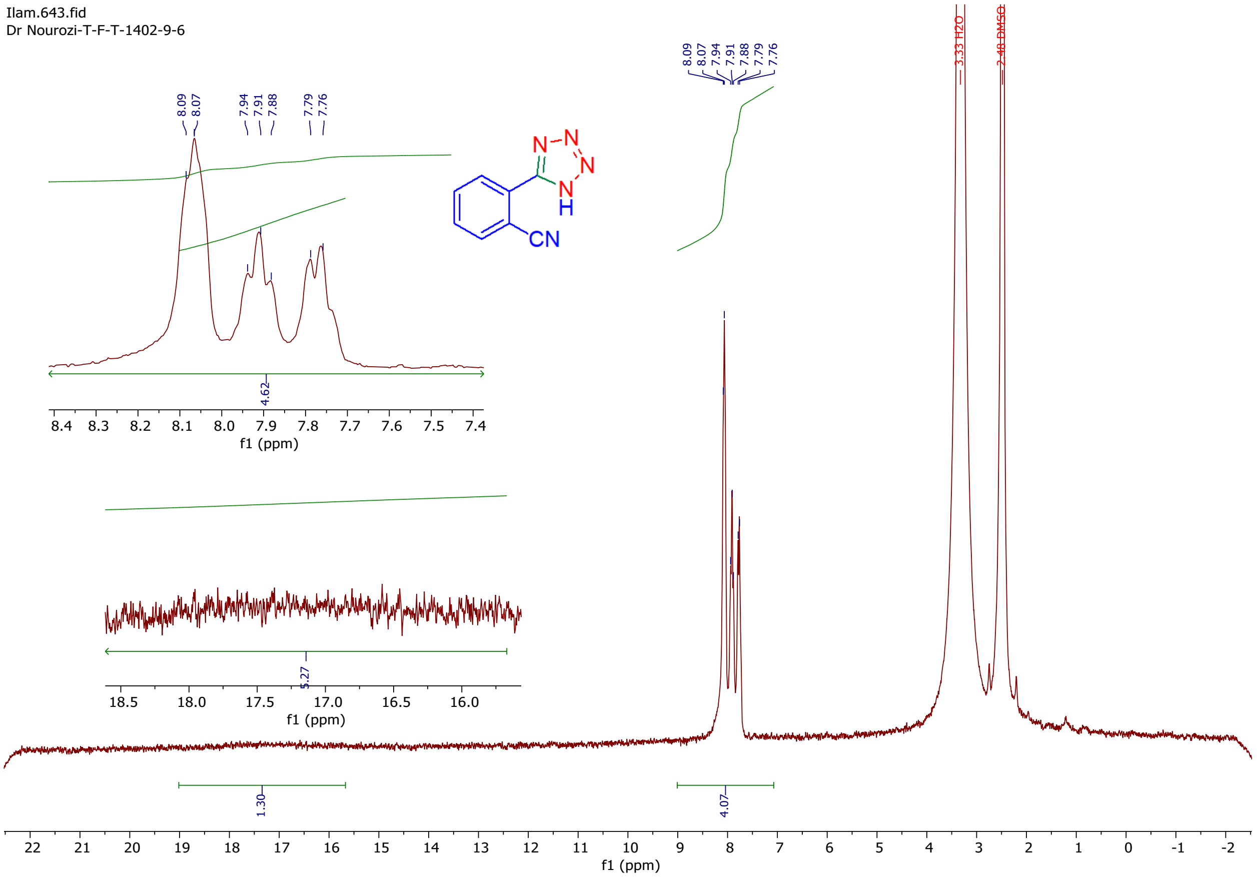

Figure S7. ^{1}HNMR spectrum of 2-(1H-Tetrazol-5-yl)-benzonitrile

2-(4-Bromo-phenyl)-2,3-dihydro-1H-quinazolin-4-one

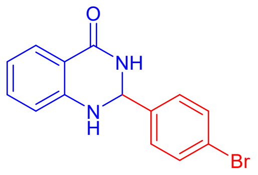

^{1}H NMR (250 MHz, DMSO-d_6) δ: 8.31 (s, 1H, NH), 7.60-7.56 (d, J= 7.6 Hz, 3H, CHAr), 7.43-7.40 (d, J = 7.4 Hz, 2H, CHAr), 7.26-712 (s, J=7 Hz, 1H, CHAr), 7.12 (s, 1H, NH), 6.70-6.66 (m, 2H, CHAr), 5.73 (s, 1H, CH) ppm.

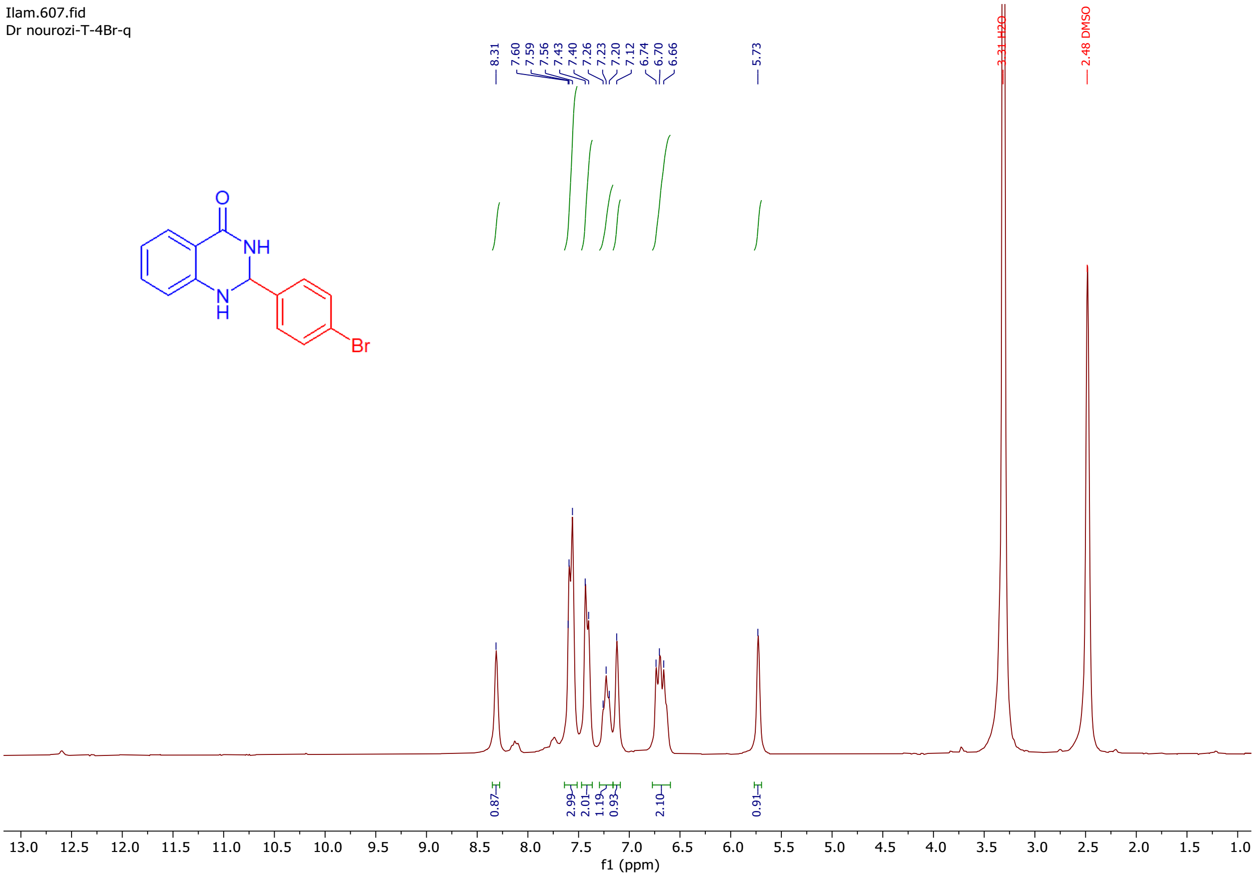

Figure S8. ^{1}HNMR spectrum of 2-(4-Bromo-phenyl)-2,3-dihydro-1H-quinazolin-4-one

2-(4-Chloro-phenyl)-2,3-dihydro-1H-quinazolin-4-one

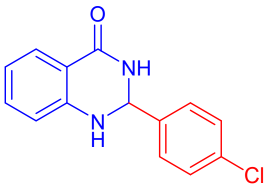

^{1}H NMR (250 MHz, DMSO-d_6) δ: 8.31 (s, 1H), 7.60-7.57 (d, J = 7.1 Hz, 1H, CHAr), 7.50-7.41 (m, 4H, CHAr), 7.26-7.19 (t, 1H, CHAr), 7.12 (s, 1H, NH), 6.73-6.66 (t, J=7.0, 2H, CHAr), 5.74 (s, 1H, CH) ppm.

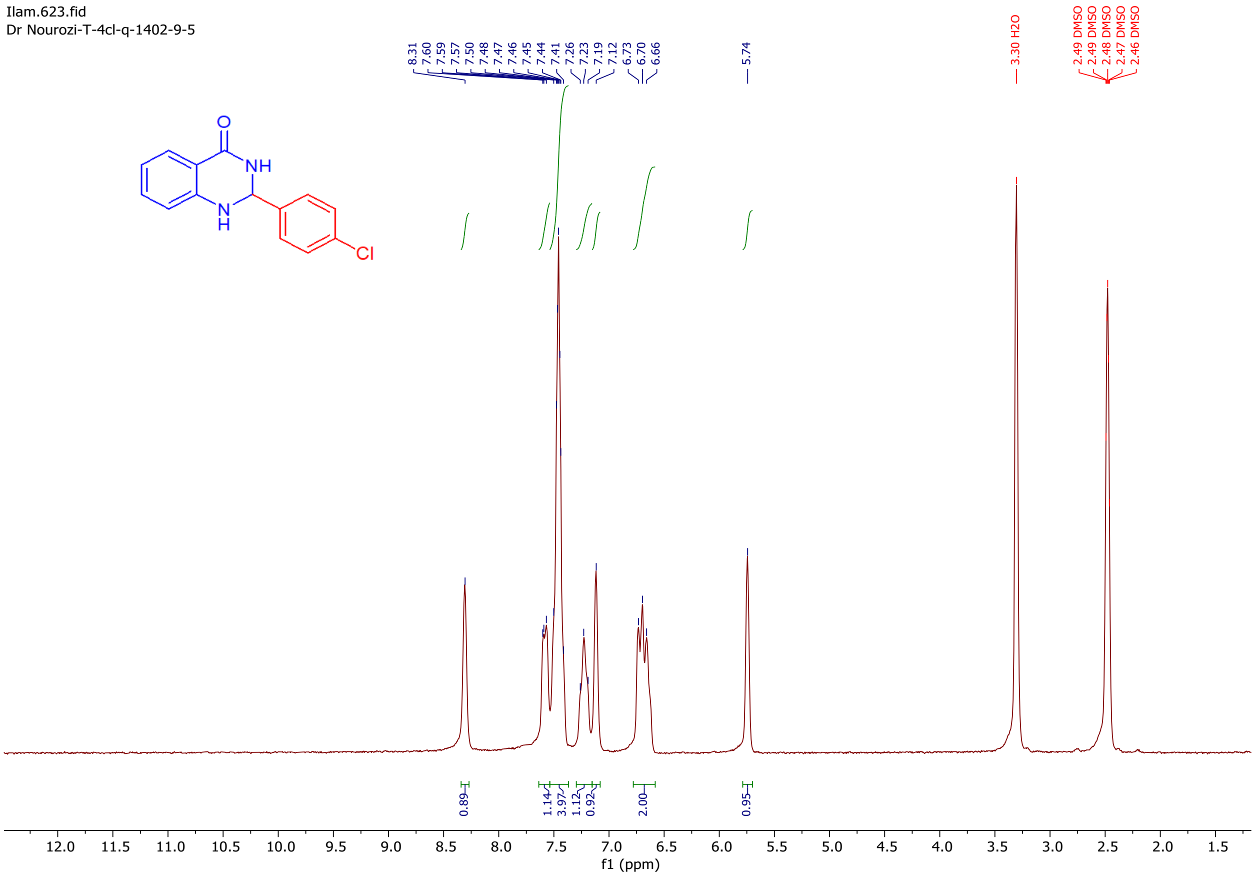

Figure S9. ^{1}HNMR spectrum of 2-(4-Chloro-phenyl)-2,3-dihydro-1H-quinazolin-4-one

2-Phenyl-2,3-dihydro-1H-quinazolin-4-one

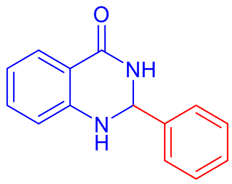

^{1}H NMR (250 MHz, DMSO-d_6) δ: 8.26 (s, 1H, NH), 7.60-7.57 (d, J = 7.6 Hz, 1H, CHAr), 7.46 (s, 2H, CHAr), 7.37-7.34 (m, 3H, CHAr), 7.25-7.19 (t, J= 7.22 Hz, 1H, CHAr), 7.09 (s, 1H, NH), 6.74-6.62 (m, J= 6.68 Hz, 2H, CHAr), 5.73 (s, 1H, CH).

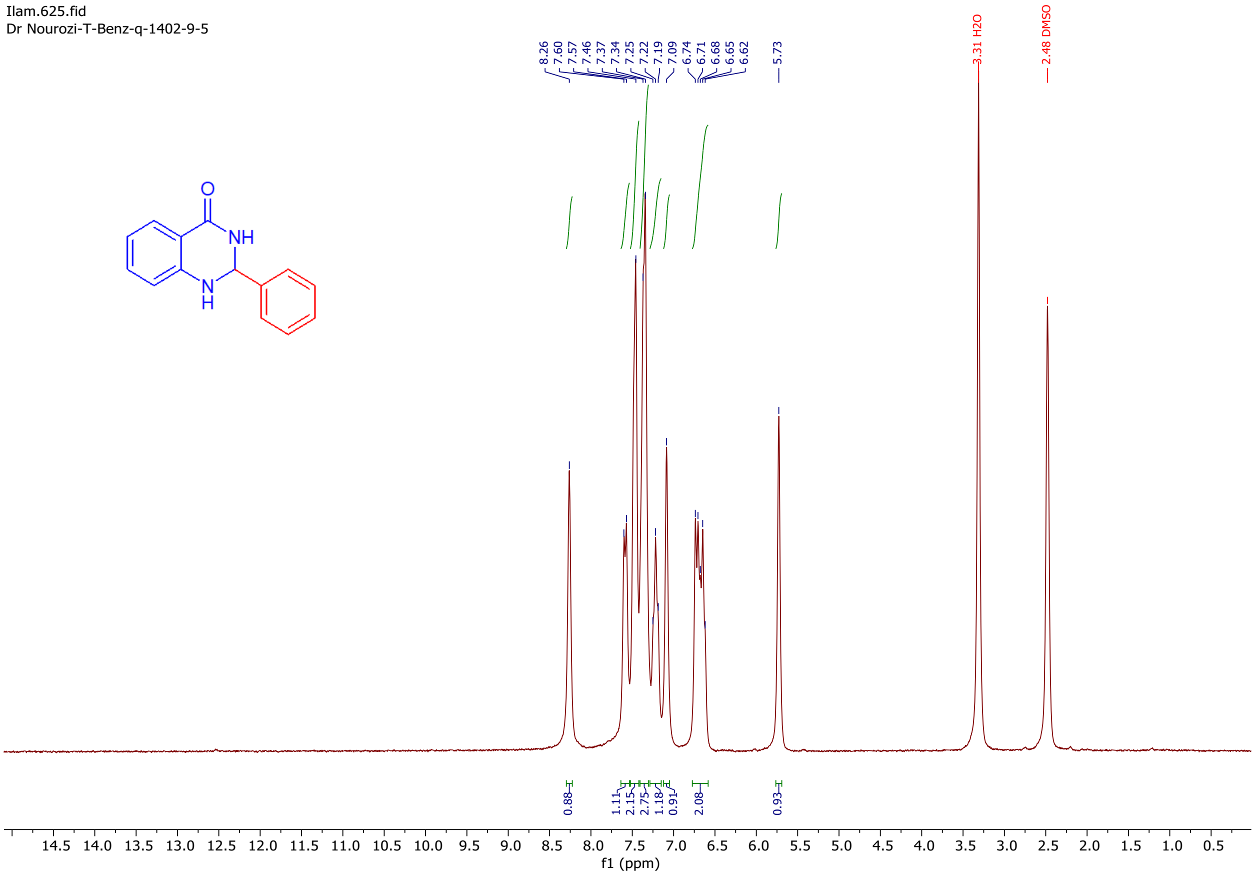

Figure S10. ^{1}HNMR spectrum of 2-Phenyl-2,3-dihydro-1H-quinazolin-4-one
